# From tradition to nutrition: wild and cultivated Aymara food plants as sustainable resources in the Andean neotropics of Bolivia

**DOI:** 10.3389/fnut.2026.1826362

**Published:** 2026-05-22

**Authors:** Simón Cocarico, Diego Rivera, Stephan Beck, Diego José Rivera-Obón, Concepción Obón

**Affiliations:** 1 Facultad de Ciencias Agrícolas Pecuarias y Recursos Naturales, Universidad Pública de El Alto (UPEA), La Paz, Bolivia; 2 Departamento de Biología Vegetal, Facultad de Biología, Universidad de Murcia, Murcia, Spain; 3 Herbario Nacional de Bolivia, Instituto de Ecología, Universidad Mayor de San Andrés, La Paz, Bolivia; 4 RITM (Research Center in Economics & Management), Université Paris-Saclay, Sceaux, France; 5 CIAGRO (Instituto de Investigación e Innovación Agroalimentario y Agroambiental), Escuela Politécnica Superior, Universidad Miguel Hernández de Elche, Orihuela, Alicante, Spain

**Keywords:** Aymara ethnobotany, Bolivian Altiplano, chuño, detoxification protocols, ethnobotany, glycoalkaloid reduction, indigenous knowledge systems, orphan crops

## Abstract

**Introduction:**

Traditional food systems of Indigenous communities embody sophisticated ecological knowledge essential for thriving in marginal environments. However, these practices have yet to be systematically documented, leaving significant gaps in our understanding of their complexity and value. In the Andean Altiplano, Aymara communities maintain complex ethnobotanical practices involving diverse food plants and specialized processing techniques, likely related with the detoxification of naturally toxic species—knowledge whose documentation is essential for understanding the role of traditional systems in sustaining agrobiodiversity.

**Objectives:**

This study characterizes the diversity of food plants and traditional processing methods used by Aymara communities in the Bolivian Altiplano, documenting the range of species and techniques employed in their food systems. It situates ethnobotanically documented food resources and processing practices within a nutrition-sensitive interpretive framework.

**Materials and methods:**

Fieldwork conducted between 2001 and 2015 combined semi-structured interviews with 288 informants—following Prior Informed Consent procedures—with participatory observation, yielding a systematically categorized dataset of plant uses and processing practices.

**Results:**

The study documented 213 food plant species from 71 botanical families, with autochthonous species accounting for 70% of all entries, underscoring the deep reliance on locally evolved biodiversity. Eight pre-culinary processing categories were identified, including alkaline ash use, freeze–thaw cycles for *chuño*, and extended fermentation for *tunta*. These methods illustrate that Aymara culinary knowledge forms a structured, empirically refined system, reflecting deep ecological and technical expertise.

**Conclusion:**

To our knowledge, this study provides the most comprehensive documentation of Aymara food ethnobotany to date, offering a taxonomically and technically detailed ethnobotanical reference. The documented diversity of food plants and processing techniques contributes to the understanding of traditional knowledge systems in climate-vulnerable highland communities. While this study does not assess household-level outcomes or nutritional intake, it establishes a foundational record of ethnobotanical practices that can inform future research and policy discussions related to agrobiodiversity and food system resilience.

## 1 Introduction

The Andes are a major center of crop domestication, where native roots and tubers have long underpinned food and nutritional security in highland communities (1, 2). While quinoa has gained global recognition as a superfood, most traditional crops cultivated by Aymara and other Andean communities remain underutilized orphan crops—a category increasingly acknowledged as critical for global food security. These species offer distinct advantages: climate resilience, high nutritional value, and adaptation to marginal environments where commercial staples fail to thrive, thereby enhancing dietary diversity and nutritional security for smallholder farmers facing agrobiodiversity loss (2, 3).

Recent advances in orphan crop genomics and breeding are accelerating their integration into global food systems, with particular potential to address nutritional gaps in low- and middle-income countries (2, 4, 5). Indigenous communities across the Andean region have acted as custodians of this agrobiodiversity for millennia, maintaining diverse crop portfolios and traditional agricultural knowledge now recognized as essential for contemporary food security.

The Aymara are an indigenous people of the Andean Altiplano, with approximately 2.3 million individuals distributed across Bolivia, Peru, Chile, and Argentina. Their agricultural systems center on locally domesticated and adapted native species [*ch‘uqi* or *papa* (*Solanum tuberosum* L. and other *Solanum* spp.), *qañawa* or *cañahua* (*Chenopodium pallidicaule* Aellen), *jupha* or *quinua* (*Chenopodium quinoa* Willd.), *apilla* or *oca* (*Oxalis tuberosa* Molina), *isañu* or *isaño* (*Tropaeolum tuberosum* Ruiz & Pav.), *k'iwicha* (*Amaranthus caudatus* L.), *ulluku* (*Ullucus tuberosus* Caldas), *racacha* (*Arracacia xanthorrhiza* Bancr.), and *tarwi* (*Lupinus mutabilis* Sweet), complemented over time by European introductions and pre-Hispanic crops from other regions of the Americas, notably maize (*Zea mays* L.)]. Over time, these traditional crops have been complemented by European introductions such as onions (*Allium cepa* L.), oats (*Avena sativa* L.), Swiss chard (*Beta vulgaris* L.), barley (*Hordeum vulgare* L.), wheat (*Triticum aestivum* L.), and broad beans (*Vicia faba* L.) (6–8).

Aymara agriculture follows a structured rotation between cultivation and extended fallow periods, closely synchronized with climatic patterns, reflecting a sophisticated land management system shaped by centuries of environmental adaptation (8, 9). The Puna ecosystems of the high Central Andes, which harbor approximately 1,500 plant species with significant nutritional and agricultural value, constitute the ecological backdrop sustaining these practices (10).

Despite their recognized importance, substantial gaps persist in the systematic documentation of Aymara food plant diversity, traditional processing methods, and associated cultural knowledge. Existing ethnobotanical studies have tended to focus on individual crops or specific localities, leaving the broader food system—and particularly detoxification protocols applied to naturally toxic species—insufficiently characterized. This gap is rendered more urgent by the ongoing nutritional transition in Andean communities, where market integration, urbanization, and dietary shift toward processed foods are eroding agrobiodiversity and weakening intergenerational knowledge transmission, while increasing diet-related chronic diseases and reducing resilience to environmental stressors (11).

This study characterizes the taxonomic diversity of wild and cultivated food plants in the Bolivian Altiplano and analyzes the traditional processing methodologies used by Aymara communities. By systematically documenting these ethnobotanical practices, we establish a foundational reference for understanding the role of traditional knowledge in agrobiodiversity conservation and the adaptation of local food systems. The manuscript does not present original mechanistic findings but rather records and interprets ethnobotanical knowledge in the context of existing scientific literature.

## 2 Materials and methods

### 2.1 Study area

**Figure 1:**
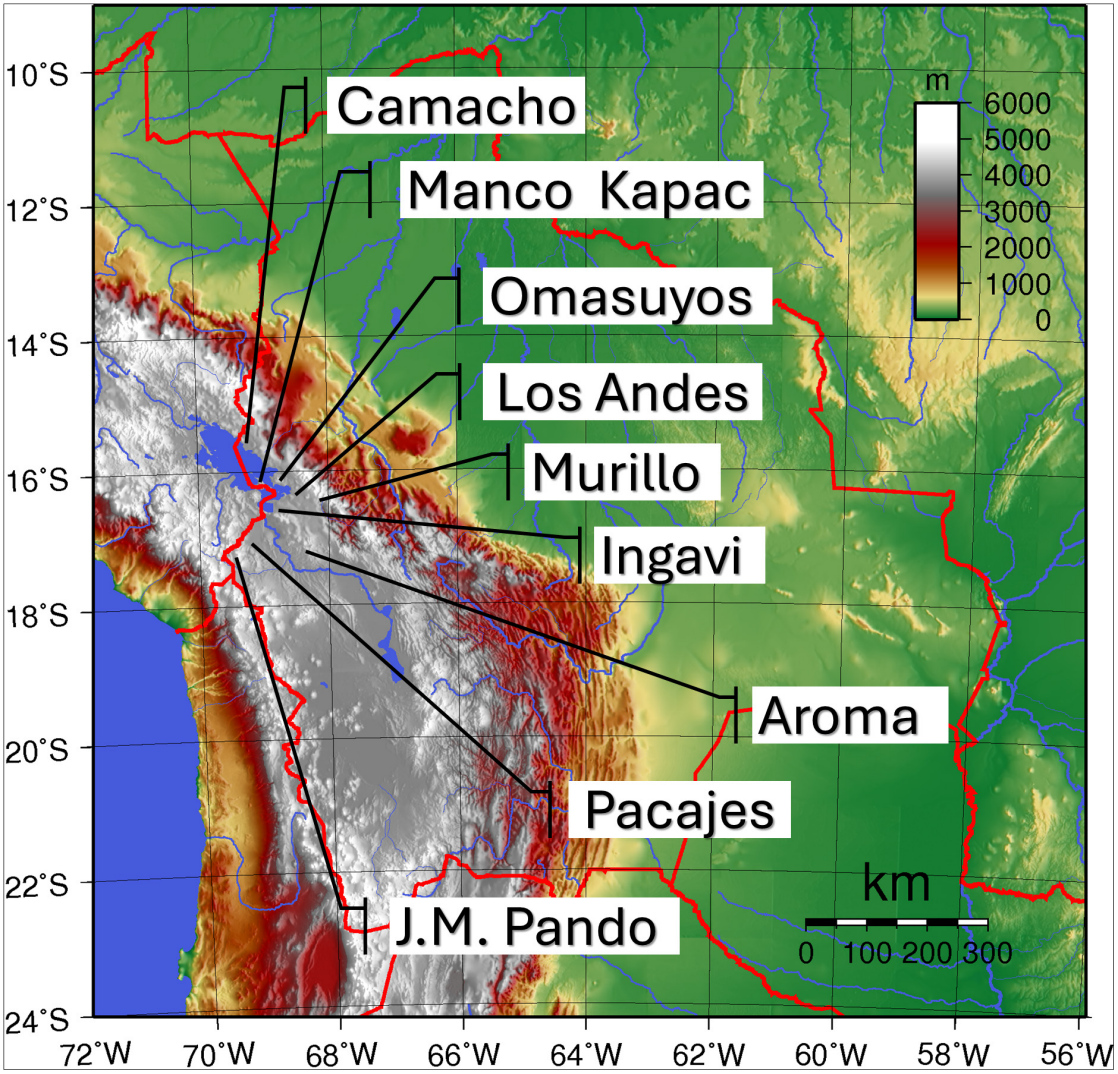
Situation and provinces of the study area in Bolivia, around the Titicaca Lake. Adapted from Sadalmelik via Wikimedia Commons, licensed under the Public Domain. Image was adapted by authors S. Cocarico and D. Rivera.

This study was conducted in Aymara communities of the La Paz Department (western Bolivia), primarily located on the Altiplano, a high-elevation plateau forming the core ecological setting of local agropastoral and foraging systems, with some participants from other Bolivian departments (Table 1; Figure 1).

**Table 1:** Informants and food entries.

Province	Relevant communities and localities	NI	%AY	FP-NI	FP-R	GR-NI	GR-R
Los Andes	Batallas, Calachaca, Catacora, Caycoma, Corapata	59	98	51	1,263	44	711
Camacho	Carabuco, Gran Puni, Puerto Acosta, Tecoaya, Ullumachi Villa Puni	51	100	46	2,546	30	744
Manco Kapac	Chañi, Copacabana, Huacuyu	39	100	32	623	24	502
Ingavi	Achaca, Calusaya, Guaraya, Huacullani, Machaca, Queruni, Rosapata, Tiahuanacu	26	100	26	853	11	228
Murillo	El Alto	19	42	10	243	1	12
Omasuyos	Corumata Alta, Hualathia, Saquena	19	95	15	425	14	225
Aroma	Calamarca, Carachuyu, Pucara	16	94	14	251	7	104
J.M.Pando	Villque	12	100	11	151	8	91
Pacajes	Pujrata	5	80	5	327	4	123
Other		42	100	22	205	2	7

Geographical provenance (except for the locations included in “Other,” the rest belong to the department of La Paz, Bolivia).

%AY, percentage of informants interviewed exclusively or predominantly in Aymara language; FP, food plants; GR, genetic resources for food and agriculture; NI, numbers of informants; R, entries.

The study area lies mainly between 3,600 and 4,100 m a.s.l., with most communities concentrated around 3,800–3,900 m. High elevation drives a cold, semi-arid climate, with mean daytime temperatures of 7–11°C and frequent diurnal frosts, particularly above 4,000 m. Annual precipitation averages ~550 mm and is strongly seasonal, with most rainfall occurring between November and February. A marked north–south gradient is observed, with higher precipitation near Lake Titicaca (~600 mm/year) and drier conditions toward the southern Altiplano (200–400 mm/year). Interannual variability linked to ENSO significantly affects water availability and plant productivity (12).

Soils are generally shallow, weakly developed, and low in organic matter. More productive Mollisols and Inceptisols occur in the northern Altiplano, supporting cultivation of staple crops such as potato and quinoa. In contrast, Aridisols and Entisols dominate the drier southern and western areas, where soils are characterized by low fertility, alkalinity, and salinization. Low water retention and high evapotranspiration further constrain plant growth (13, 14).

Vegetation consists primarily of high-altitude grassland and shrubland. Dominant species include *ichu* (*Jarava ichu* Ruiz & Pav.), *tola* (*Parastrephia* spp.), and cushion plants such as *yareta* (*Azorella compacta* Phil.). In wetter areas near Lake Titicaca, totora (*Schoenoplectus californicus* (C.A.Mey.) Soják) forms extensive stands, while remnant native trees (*Polylepis* spp., *Buddleja coriacea* J.Rémy) persist in protected sites. Vegetation becomes increasingly sparse toward the arid southern Altiplano (10, 15).

The region encompasses a strong environmental gradient in moisture availability, soil quality, and vegetation structure. These factors directly influence the distribution, diversity, and management of cultivated and wild food plants, shaping Aymara ethnobotanical knowledge and subsistence strategies (8).

### 2.2 Ethnobotanical surveys

We employed ethnographic methods combining long-term fieldwork (2001–2015), semi-structured interviews with 288 informants, and participatory observation. Fieldwork involved sustained community integration through residence and collaborative engagement at the primary study sites. All interviews were conducted in accordance with Prior Informed Consent (PIC) procedures, following verbal consent protocols consistent with the ISE Code of Ethics (16); verbal rather than written consent was employed in accordance with standard practice in ethnobotanical research with indigenous communities, where written instruments can be culturally inappropriate or conflict with oral tradition norms. Regarding participants under 18 years of age, all interviews involving minors were conducted following appropriate ethical protocols. In every case, participation required both the assent of the minor and the informed consent of a parent or legal guardian. Participant data were anonymized and handled in accordance with applicable data protection standards. Formal community-level ethical authorization was granted by the local Aymara community council (*ampliado cantonal*) of Villa Puni, which constituted the recognized governance body for research approval in the primary study area of Villa Puni, Gran Puni, and Ullumachi, Camacho Province. Additional data were recorded in the provinces of Los Andes, Manco Kapac, and Ingavi, with supplementary research conducted in Murillo, Omasuyos, and Aroma. At the time of the earliest fieldwork, formal permission from local authorities was not required for research conducted outside the central study area. Most of the disparities shown in Table 1 are due to the availability of time and resources for interviewer, longer periods of residence, or repeated contact with specific communities.

All interviews followed a standardized protocol, with Aymara as the predominant language of communication. Of the 288 informants interviewed, 159 (55.2%) conducted their interview entirely in Aymara, 101 (35%) in predominantly Aymara but combined with Spanish, and 28 (9.7%) in Spanish only. The interviews covered a very broad field within ethnobotany. However, in this study, of the 288 informants, only 232 spoke about food, and of these, 145 provided details about its cultivation (Table 1).

The names of plant species and varieties were recorded and retained in the original language or languages of the interview, as were the designations of the preparation processes to which the plants were subjected and the resulting dish or food type.

The interviews were recorded in the field notebook in the language in which they took place. During the process of entering the data into the database, the various uses and processing methods recorded for each taxon (species, subspecies, and varieties), plant part, and each informant were organized as separate entries (records). Proportionally registered entries were higher from interviews in Aymara combined with Spanish (50.9%), followed by Aymara (47.2%) and very few from those in Spanish (1.8%). Most of the informants were interviewed only once (184) or twice (54). In the cases of repeated interviews saturation was reached when repeated interviews yielded no novel taxa or processing methods. Detailed descriptions of foods and preparation processes were drawn from accounts provided by the informants, complemented from direct observation by the researcher during the interviews, where food was processed; these detailed descriptions were subsequently incorporated into the database in Spanish, as translated by one of the authors (SCY). Local markets were chosen as suitable locations for gathering information on edible plants, using the same interview protocol, this time with vendors, where the botanical identification of the specimens proved easier.

Visual documentation of plants, ceremonies, markets, farms, and community events was conducted by team member Simón Cocarico.

Plant specimens were collected systematically, including the species mentioned by informants—whether those suggested by them or those identified through direct observation in various ecological zones, notably cultivated plots, fallow land, and abandoned fields spanning several hundred hectares, generally in the presence of an informant to verify that these specimens corresponded to the plants used locally. Specimens were collected, pressed, and dried following standard herbarium protocols. Ethnobotanical data prioritized *in situ* documentation of local plant names with informants, although some taxa required subsequent laboratory identification. The protocol was expanded to include market-sourced specimens from Altiplano markets to document commercialized ethnobotanical resources mentioned by vendors.

### 2.3 Informant demographics

The study included 288 informants selected through purposive sampling of key knowledge holders (e.g., rural housewives and experienced farmers). The sample was demographically diverse, with near gender parity and ages ranging from 14 to 100 years (mean = 46.7), and most participants engaged primarily in agriculture, often combined with secondary occupations (Table 2).

**Table 2:** Distribution of informants, recorded food-use entries, mean age by profession, and female and male proportions.

Profession	Informants (*n*)	Food-use entries (*n*)	Mean age (years)	Female	Male
Farmer	154	4,582	52.5	56	98
Student	36	1,959	23.8	16	20
Vendor	34	135	41.7	25	9
Craftsman	9	69	53.4	1	8
Housewife	30	763	54.5	30	0
School teacher	3	139	26.3	2	1
Religious leader	4	202	40.0	1	3
Agronomist	4	35	29.3	3	1
Cook	3	153	46.7	3	0
Mechanic	1	17	40.0	0	1
Livestock technician	2	272	36.0	0	2
Dairy farmer	6	81	30.3	3	3
Driver	1	0	45	0	1
Baker	1	0	55.0	0	1
Total	288	8,407	—		

Fieldwork was conducted during ecologically and culturally significant periods, including key climatic seasons and harvest-related festivities. Data collection combined direct observation of household practices, community activities (collective labor, ceremonies, and religious events), and systematic observations of weekly markets documenting plant trade between Altiplano and Valley populations.

Research was intensive and long-term, involving at least 3 months of fieldwork annually during 2001–2003 and continued engagement, thereafter, totaling over 100 site visits. Sustained immersion by one researcher with cultural ties to the Aymara community provided additional depth through participatory observation.

### 2.4 Taxonomic identification

Voucher specimens were deposited at the Herbario Nacional de Bolivia (LPB), Universidad Mayor de San Andrés, La Paz. Field data were recorded in notebooks and digitized into Excel workbooks organized by informants, locations, medicinal plants, germplasm, agriculture, food, fuels, and forages. For the present study, analyses focused on informant, food plant, and germplasm datasets. Plant nomenclature was standardized using the *Catálogo de las Plantas Vasculares de Bolivia* (17) and POWO (18). Species (vascular, algae, cyanobacteria) were verified at the Catalog of Life and GBIF (19–21).

### 2.5 Data analysis and visualization

We categorized “Life Forms” (or Plant type) and “Provenance” (autochthonous, introduced, imported, based on the distribution data) for the species, according to POWO (18). Instead “Status” (wild/cultivated), and “Mode of Acquisition” (exchange/market) resulted primarily from the interviews with the informants.

In this study, a “record” or “entry” refers to a coded use-report, systematically structured by taxon, plant part, preparation or process, and informant. This granular approach is a standard methodological practice that enables the documentation of multi-functional taxa and context-specific uses. Consequently, the resulting counts do not represent statistically independent “events,” but instead reflect the cumulative documentation of knowledge elements reported by informants across diverse use contexts.

Basic statistics were performed using the different tools available in Excel 365 and in R (Version 4.5.2, R Core Team) (22). The use of descriptive statistics is appropriate for this type of dataset, particularly given its uneven structure, the diversity of contexts, and the absence of a sampling design that would support robust inferential or model-based approaches without introducing additional assumptions.

Two datasets were used for analysis. The first documents associations between plant type (botanical/culinary), plant species, and food detoxification processes; the second documents associations between plant species and traditional Aymara dishes. Both datasets are quantified by ethnographic citation counts per combination. For each plant species, the proportion of citations distributed across dishes was calculated; conversely, for each dish, the proportional contribution of each plant was computed. These relative measures complement absolute citation counts and allow structural patterns in plant–food associations to be examined independently of overall citation volume.

Relationships in both datasets were visualized using alluvial plots constructed with the *ggalluvial* package (23, 24) in R (Version 4.5.2, R Core Team) (22) within the *ggplot2* ecosystem (25). Alluvial plots represent multi-categorical flows, where vertical strata correspond to categorical variables (e.g., plant species, dish) and connecting bands (alluvia) are scaled proportionally to a quantitative variable: flows represent aggregated counts of coded records (use-report tables) linking taxa, processing categories, and other variables; a full data dictionary and input matrices are provided in GitHub (https://github.com/drivera2001/Aymara-Food). The diagrams are exploratory and relational, aimed at making visible how plant materials, processing practices, and use contexts co-occur within reported knowledge systems.

For the first dataset, a three-dimensional alluvial plot connected plant species, detoxification processes and plant types, across three axes. To reduce skewness and improve the visibility of secondary flows, citation counts were log-transformed. For the second dataset, two-dimensional alluvial plots linked plant species to dishes. Two specifications were produced: one based on absolute citation counts, highlighting species and dishes of greatest overall cultural salience, and one using dish-level proportional measures, controlling for differences in overall dish popularity and emphasizing compositional variation in botanical inputs across dishes.

## 3 Results

### 3.1 Gender and age dynamics

Farmers and students contributed the majority of informants and food-use records, with notable variation in contributions across professions (Table 2).

Our observed patterns, rather than formally tested relationships, suggest that women informants—who were, on average, younger than their male counterparts (43 vs. 48.9 years)—contributed a smaller total number of entries (3,497 vs. 4,910) with an average number of entries per informant of 15.1 vs. 19.9, and demonstrated lower proportional coverage across cooking and pre-culinary processing (41 vs. 58% in both, a 17% gap), stages that encompass labor-intensive tasks such as ingredient gathering, selection, ash preparation (e.g., *llujt'a* alkaline processing), and kneading. In the final dish stage, women maintained a slight but consistent advantage (51 vs. 48%). These percentages represent the proportion of entries where apart from the local name of the plant were registered specific uses or processing techniques, or even although into a much lesser level, local dishes.

These results are clearly not conclusive and contrast with ethnographic observations of Aymara gender roles, wherein women traditionally oversee the transformative, daily processes critical to food safety and household sustenance—including tuber detoxification, storage preparation, and the application of specialized techniques like alkaline treatment (11). Rather than indicating a rigid divide, the findings suggest a complementary, overlapping knowledge system (8).

**Figure 2:**
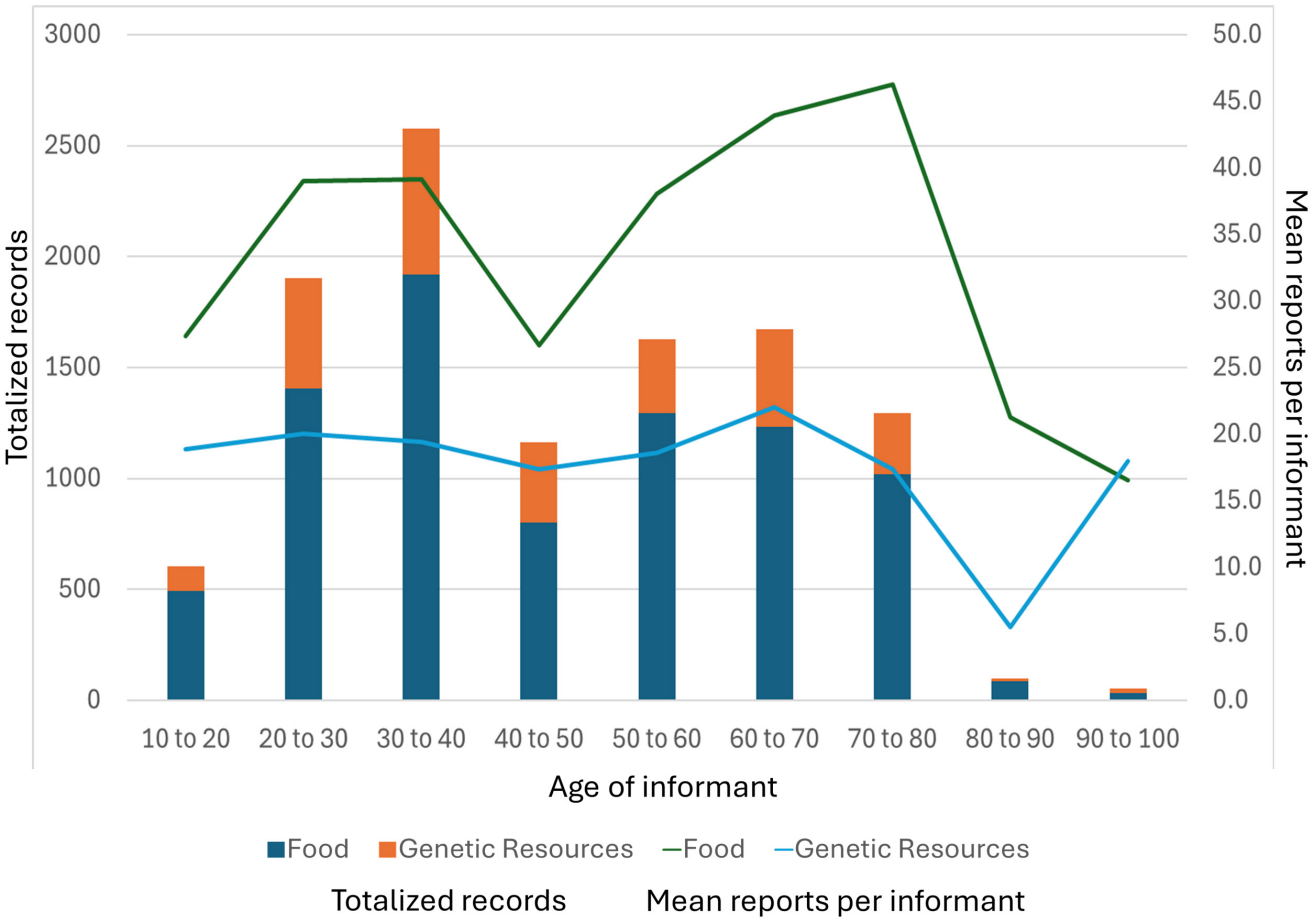
Distribution of knowledge of food plants and their role as genetic resources across age categories among 288 Aymara informants, showing total entries and mean entries per informant. The pattern highlights age-related differences in knowledge accumulation and transmission, with lower values in middle-aged cohorts and higher individual knowledge among younger adults and elders.

Concerning age, the graphic (Figure 2) shows an age-related bimodal pattern in the knowledge of food plants and their role as genetic resources. Overall, the results suggest, based on observed patterns rather than formally tested relationships:

- Progressive accumulation of ethnobotanical knowledge over the life course, which, on average, reach a maximum at an age of c. 80 (Figure 2), closely tied to long-term participation in farming, seed management, and food practices.
- Smaller knowledge among younger informants, reflecting processes such as schooling outside the community, market integration, migration, and declining everyday engagement with traditional agriculture.
- A potential intergenerational erosion of knowledge related to food plants as genetic resources, which raises concerns for the continuity of local agrobiodiversity management.

The lower scores observed among informants aged 40–60 suggest, but not proves, a generational discontinuity in the transmission of knowledge, associated with increased engagement in wage labor, formal education, and agricultural modernization during their formative years. The two peaks in mean entries (records) per informant, at 30–40 and 60–80 years, reflect contrasting life-course dynamics: renewed engagement with traditional food-plant knowledge during household formation and the role of elders as long-term custodians of agrobiodiversity. Overall, the non-linear age distribution highlights socially mediated breaks and partial recoveries in knowledge transmission rather than a simple accumulation of knowledge with age. However, the observed age patterns could potentially have been influenced by recruitment structure (e.g., differential access to students vs. long-term community members, interviewer presence, and engagement with specific groups), rather than as direct evidence of knowledge erosion. We acknowledge that a more robust assessment of intergenerational change would require inferential modeling incorporating covariates such as locality, occupation, gender, and recruitment strategy.

### 3.2 Taxonomic breadth

The taxonomic distribution of 213 species and subspecies, 227 taxa including cultivar groups, across 71 plant families, reveals pronounced taxonomic concentration alongside substantial diversity. Solanaceae exhibits overwhelming dominance with approximately 2,000 entries representing 24 taxa (~12% of total species diversity), reflecting the central importance of potato (*Solanum tuberosum* and related Andean tuber species) in traditional highland agriculture. This family alone accounts for nearly one-quarter of all entries.

**Figure 3:**
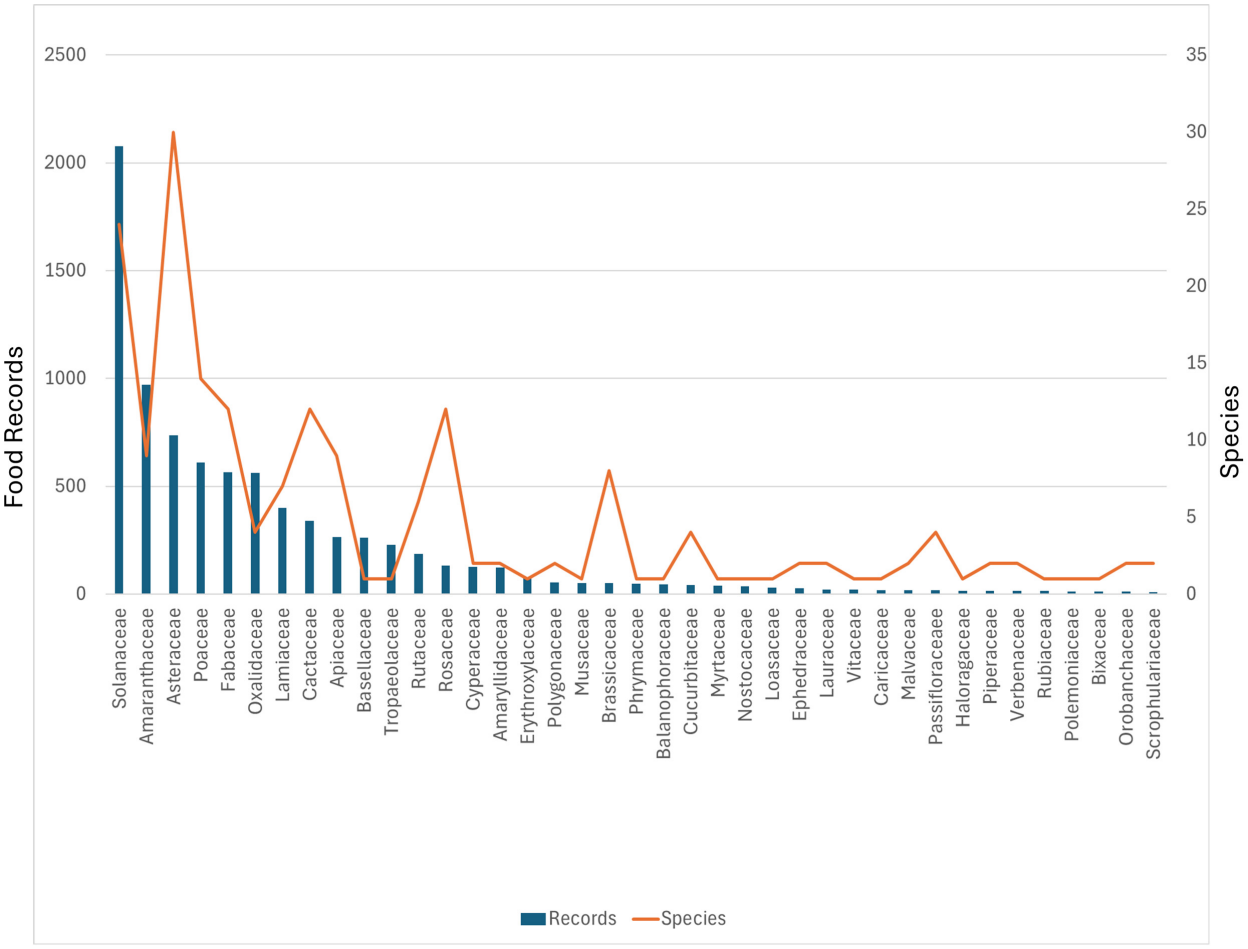
Distribution of plant entries and species richness across taxonomic families. Blue bars represent number of entries (left y-axis); orange line shows number of species (right y-axis). Solanaceae dominates with approximately 2,000 entries and 24 species, followed by Amaranthaceae, Asteraceae, and Poaceae. Plant families with less than 10 entries in the database are not represented in the figure.

The subsequent families—Amaranthaceae (with notably *Chenopodium quinoa*, formerly included in Chenopodiaceae) (19), Asteraceae, and Poaceae—show moderate representation with 600–900 entries each and 9–30 species, demonstrating both agricultural significance and natural floristic diversity. A sharp decline follows, with most families (approximately 50 of the 71 families) contributing fewer than 100 entries each, yet collectively harboring substantial species richness. This pattern is evident in the orange trend line, which maintains relatively stable values (1–3 species per family) across the long tail of underrepresented families (Figure 3).

**Figure 4:**
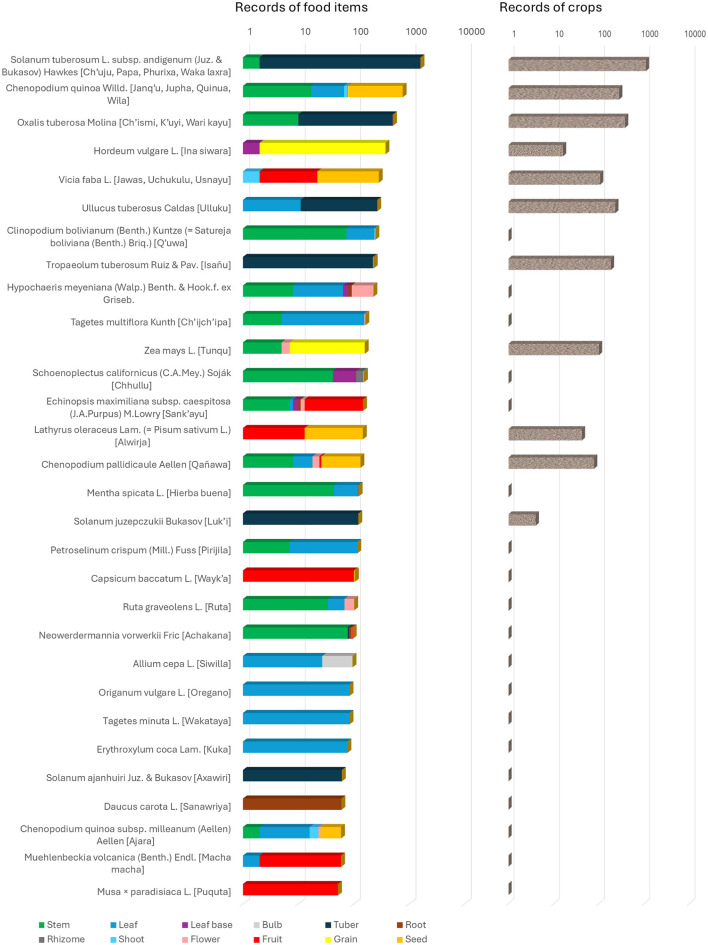
Comparison of information richness between food use (left panel) and agricultural management (right panel) for the most frequently cited plant species in Aymara communities of *Departamento de La Paz*, Bolivia. Species are ranked by total food-related citations on a logarithmic scale. The left panel displays cumulative citations related to food applications, including processing methods, and culinary preparations for plant parts used as ingredients in traditional dishes (color-coded by plant part: stem, leaf, base, bulb, tuber, root, rhizome, shoot, flower, fruit, grain, and seed). The right panel presents, on a logarithmic scale, citations of the same species concerning cultivation practices, varietal diversity, and agronomic knowledge related to these species as genetic resources. Disparities between panels reveal differences in the cultural emphasis placed on species as food resources vs. managed crops. After the scientific name of each plant are recorded between square brackets the more frequent Aymara names. Notice: 165 species with 50 entries or less are not comprised in this figure which only includes the 30 species more frequently cited. Figure was created by D. Rivera.

The 71 families (Figure 3) encompass the full spectrum of plant life forms documented in the region, from dominant agricultural species to occasional wild-gathered resources, medicinal plants, and ritual species, that share their primary value as food. The distribution in Figure 4 reflects the dual nature of the dataset: intensive documentation of economically critical crop species (particularly tuberous geophytes) combined with broader sampling of wild and semi-domesticated taxa utilized in traditional Andean subsistence systems.

The dataset exhibits pronounced dominance of Andean tuberous geophytes, with *Solanum tuberosum* subsp. *andigenum* overwhelmingly representing the most documented taxon (1,629 entries, 19% of total; Figure 4). This reflects the centrality of native potato diversity in highland subsistence, with secondary tuber crops including *Oxalis tuberosa* (515 entries), *Ullucus tuberosus* (261 entries), and *Tropaeolum tuberosum* (229 entries) collectively accounting for an additional 13% of entries. Frost-resistant potato taxa requiring specialized detoxification processing—*Solanum juzepczukii* (124 entries), *S. ajanhuiri* (61 entries) and *S. curtilobum* (39 entries)—represent important genetic resources for high-altitude cultivation and freeze-drying technologies. The pseudocereal *Chenopodium quinoa* constitutes the second-ranking taxon (758 entries, 9% of total), with its high-altitude ecotype *C. quinoa* subsp. *milleanum* (54 entries) and the cold-tolerant *cañihua* (*C. pallidicaule*, 119 entries) providing complementary grain sources adapted to conditions unsuitable for introduced cereals.

**Figure 5:**
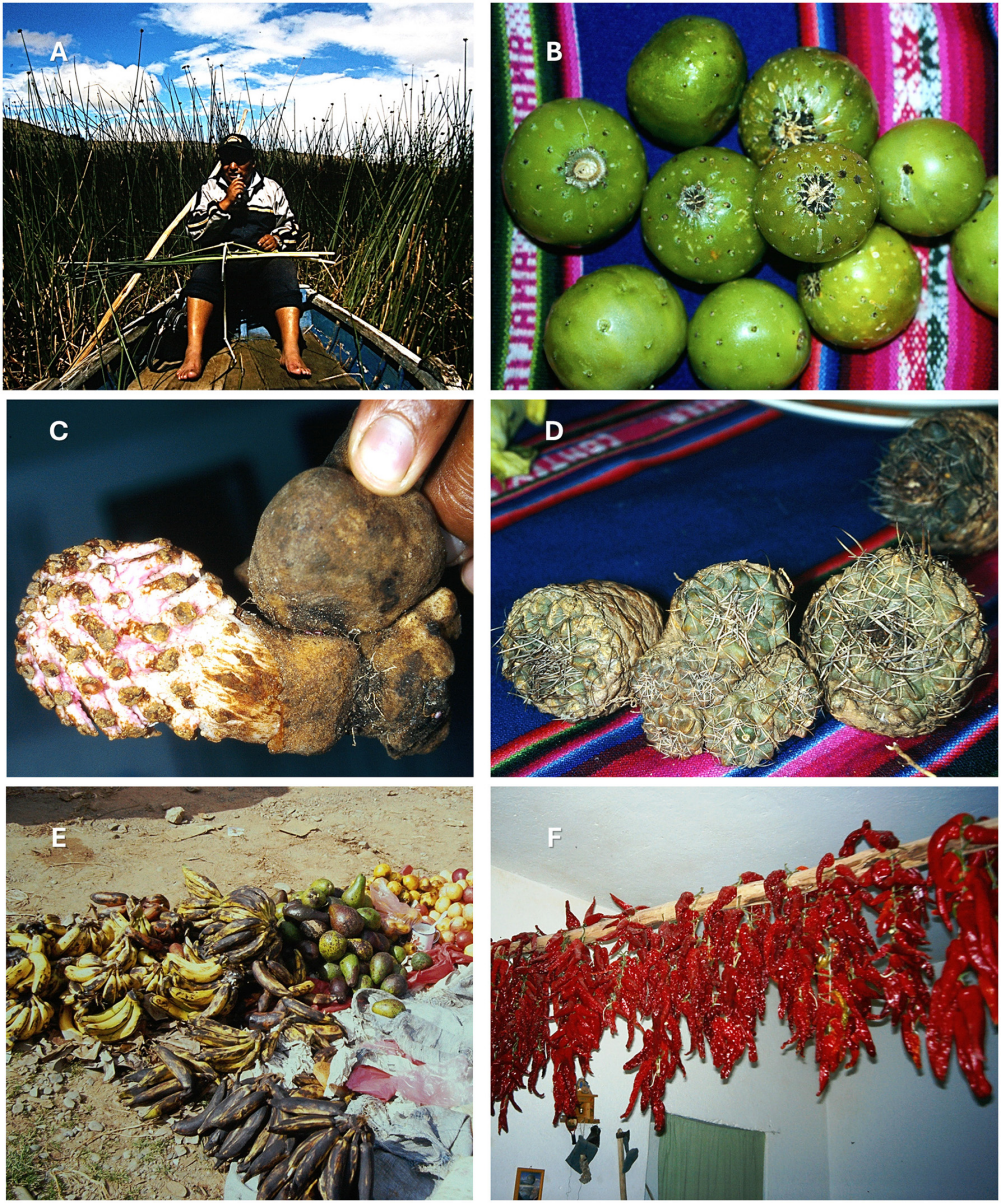
Traditional minor foods from Aymara communities of the Bolivian altiplano. **(A)** Harvesting of totora (*Schoenoplectus californicus*) from lake wetlands, demonstrating traditional methods for extracting and consuming the basal portion of the tutura stem. **(B)** Fresh fruits of *Corryocactus melanotrichus* (K.Schum.) Britton & Rose (*jawaq'ulla*) displayed on traditional Andean textile. **(C)** Edible *Ombrophytum subterraneum* (Aspl.) B.Hansen (*amañuqi*), parasitic plant from Balanophoraceae. **(D)** Dried and bundled cactus stems, *Neowerdermannia vorwerkii* Fric. (*achakana*), prepared for storage and consumption. **(E)** Local market display featuring various produce including *Musa* sp. fruits (*plátano*) and other regional cultivars. **(F)** Sun-dried *Capsicum baccatum* L. (*wayk'a*) peppers hanging for preservation.

Introduced crops demonstrate substantial integration into traditional systems, with barley (*Hordeum vulgare*, 373 entries) and broad bean (*Vicia faba*, 280 entries) ranking fourth and fifth respectively, while maize (*Zea mays*, 152 entries) and peas (*Lathyrus oleraceus*, 146 entries) contribute moderate frequencies. Non-food crops occupy significant positions, including the aromatic subshrub *Clinopodium bolivianum* (133 entries) also used medicinally, coca (*Erythroxylum coca*, 77 entries) serving ritual and stimulant functions, and the totora reed *Schoenoplectus californicus* (113 entries) providing edible rhizomes (Figure 5). Wild-gathered taxa maintain persistent representation, particularly *Hypochaeris meyeniana* (220 entries), a leafy vegetable—of which the edible portion consists of the flower bud (*mut'isu*), specifically the tender flowers, the involucral bracts, and the leaves—subjected to ash processing for detoxification, and cactuses *Echinopsis maximiliana* subsp. *caespitosa* (144 entries), whose fruit is consumed, and *Neowerdermannia vorwerkii* (*achakaha*; 96 entries), from which the pulpy tissue of its fleshy stem is eaten after removing the spines and peeling the star-shaped epidermal ridges (Figure 5) providing emergency foods and ritual offerings, demonstrating continuity of wild resource exploitation alongside intensive agriculture.

Some among the 213 species, notwitstanding their relevance as food or condiment, were less often recorded: that is, *Cuminum cyminum* L. (47 entries), *Ombrophytum subterraneum* (Aspl.) B. Hansen (*amañuqi*; 44 entries; Figure 5), *Erythranthe glabrata* (Kunth) G.L.Nesom (= *Mimulus glabratus* A.Gray, 48 entries), *Solanum lycopersicum* L. (39 entries), *Nostoc commune* Vaucher ex Bornet & Flahault (37 entries), *Lepechinia meyenii* (Walp.) Epling (36 entries), *Ageratina glechonophylla* (Less.) R.M.King & H.Rob. (32 entries), *Cucurbita maxima* Duchesne (25 entries), *Corryocactus melanotrichus* (K.Schum.) Britton & Rose (12 entries; Figure 5), and others.

**Figure 6:**
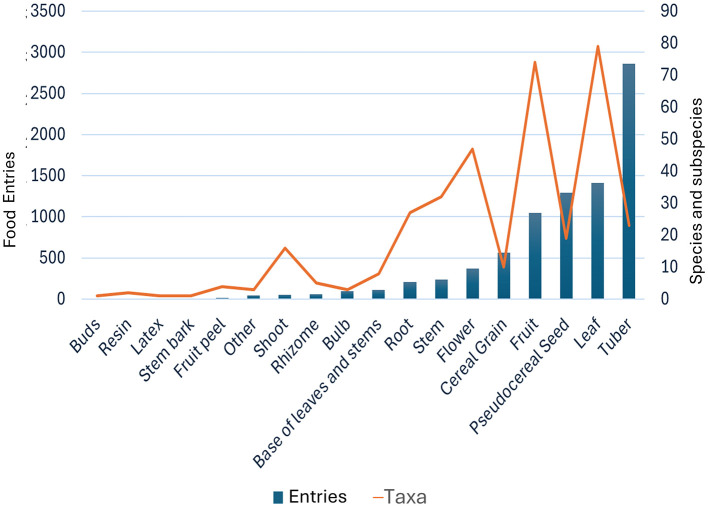
Utilization patterns by plant part category. Bars: number of entries (left axis); line: number of taxa per plant part (right axis). Categories ranked by record abundance. Tubers show highest record frequency, reflecting the importance of tuberous geophytes in Andean agriculture, followed by leaves and pseudocereal seeds. The orange line reveals high taxonomic diversity in leaf and tuber categories despite varying record frequencies.

Figure 6 illustrates the distribution of 8,000+ entries across 15 plant part categories, revealing utilization priorities and taxonomic diversity in traditional Andean food systems. The data demonstrates pronounced concentration on specific plant parts while maintaining taxonomic breadth across categories.

**Figure 7:**
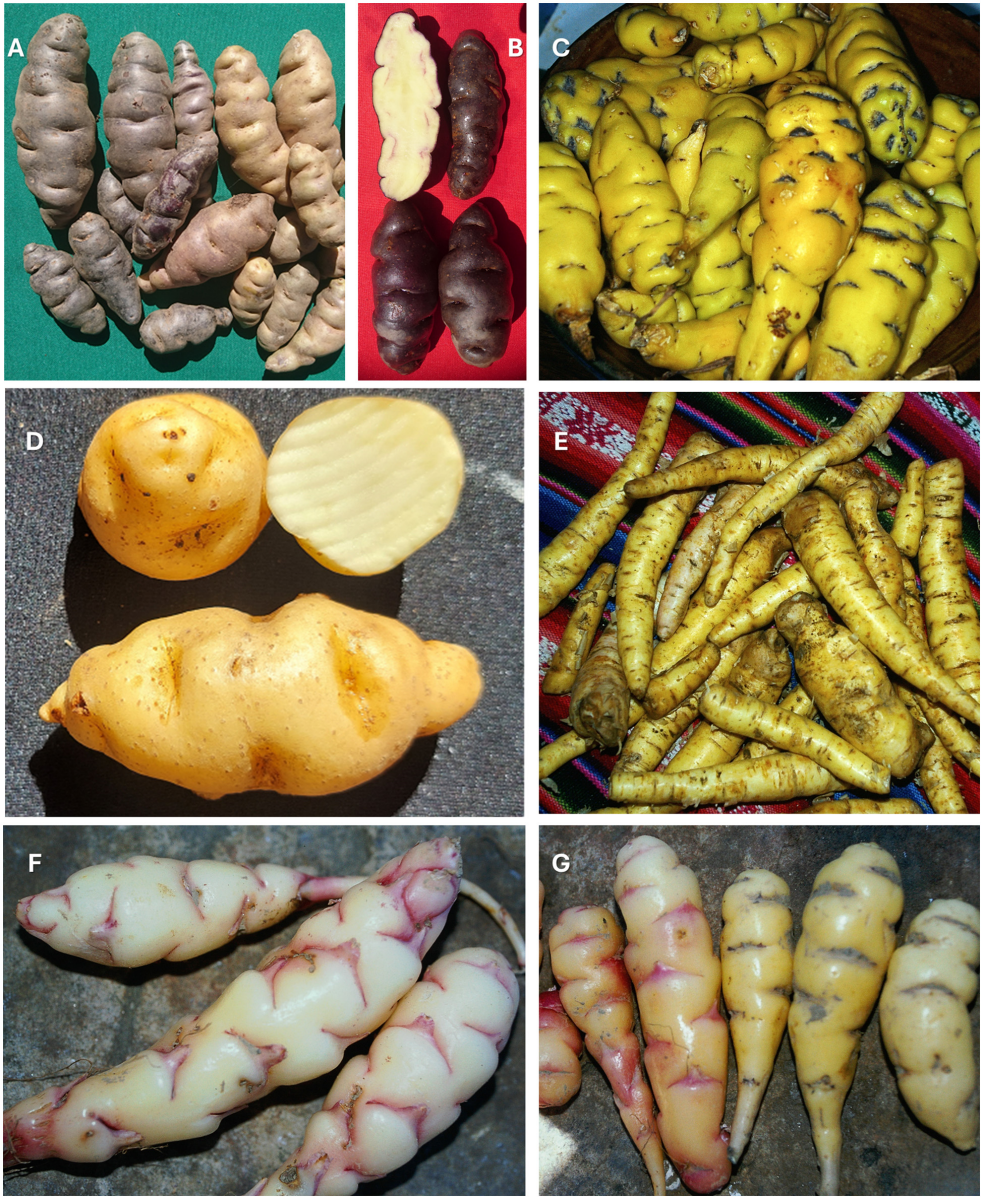
Diversity of traditional Andean tuber crops from Aymara communities of the Bolivian altiplano. **(A, B)** *Solanum ajanhuiri* (*axawiri*) showing characteristic smooth, elongated tubers with varied pigmentation. **(C)** *Tropaeolum tuberosum* (*isañu*), displaying the distinctive, yellow-pigmented landrace with purple markings. **(D)** *Solanum ajanhuiri* (*ch'uqipitu*), native Andean potato, tubers. **(E)** *Arracacia xanthorrhiza* (*arracacha/racacha*) showing the characteristic elongated, cylindrical yellow roots. **(F)** *Oxalis tuberosa* (*oca*) displaying white tubers with characteristic red-purple striations and irregular knobby surface. **(G)** Morphological comparison between *Oxalis tuberosa* (left, more conical) and *Tropaeolum tuberosum* (right, elongated with gray striations), illustrating interspecific variation in tuber shape and pigmentation patterns. These underutilized Andean tuber species represent significant agrobiodiversity and provide essential nutritional and cultural value in traditional highland food systems.

Tubers dominate overwhelmingly with approximately 2,859 entries representing 23 taxa, underscoring the centrality of tuberous geophytes—particularly potato (*Solanum* spp.) diversity—in highland Andean subsistence (Figure 7). This single category accounts for one-third of all entries, reflecting both the abundance of tuber crops and the intensive documentation of traditional processing methods for storage crops (*chuño, tunta*, and fresh preparations).

Leaves constitute the second major category with 1,415 entries across 79 taxa, demonstrating higher taxonomic diversity to tubers but lower record frequency. This reflects the use of diverse leafy vegetables, herbs, and aromatic plants for culinary purposes, ranging from cultivated greens to wild-gathered species. Some of these plants are also used as medicinal resources (8).

Pseudocereal seeds (primarily quinoa and *cañihua*) show approximately 1,300 entries with moderate taxonomic diversity (19 taxa), representing the critical grain component of highland diet adapted to high-altitude conditions where true cereals perform poorly. The high record count reflects quinoa's processing versatility across multiple food preparations.

Cereal grains (barley, maize, wheat) exhibit high record frequency (565 entries) but moderate taxonomic diversity (10 taxa), indicating intensive use of a limited number of staple grain species. Fruits show approximately 1,000 entries with exceptionally high taxonomic diversity (74 taxa), suggesting broad utilization of diverse fruit species, though individual species may be used less intensively than staple crops.

Flower (374, 47), stem (234 entries, 32 species), root (209, 27), and base of leaf (112, 8) categories show progressively lower record and taxa counts, representing specialized uses of these plant parts across diverse species. The orange trend line reveals that despite lower record frequencies, these categories maintain substantial taxonomic diversity, indicating that many species contribute specific plant parts to the traditional food system.

Minor categories including bulbs (99 entries, 3 species), rhizomes (60, 5), shoot (50, 16), fruit peel (15, 4), stem bark (9, 1), latex (7, 1), resin (6, 2), and buds (2, 1) show minimal entries but collectively represent specialized knowledge about utilizing secondary plant parts. The sustained presence of 1–10 taxa per category demonstrates the comprehensiveness of traditional ethnobotanical knowledge extending beyond primary food organs. Finally, a minor category designated “other” (44 records, 3 taxa) includes non-vascular organisms—specifically cyanobacterial and algal biomass—that, while taxonomically distinct from vascular plants, constitute traditional food sources integrated within Andean subsistence systems.

### 3.3 The origin and provenance of Aymara plant food

**Figure 8:**
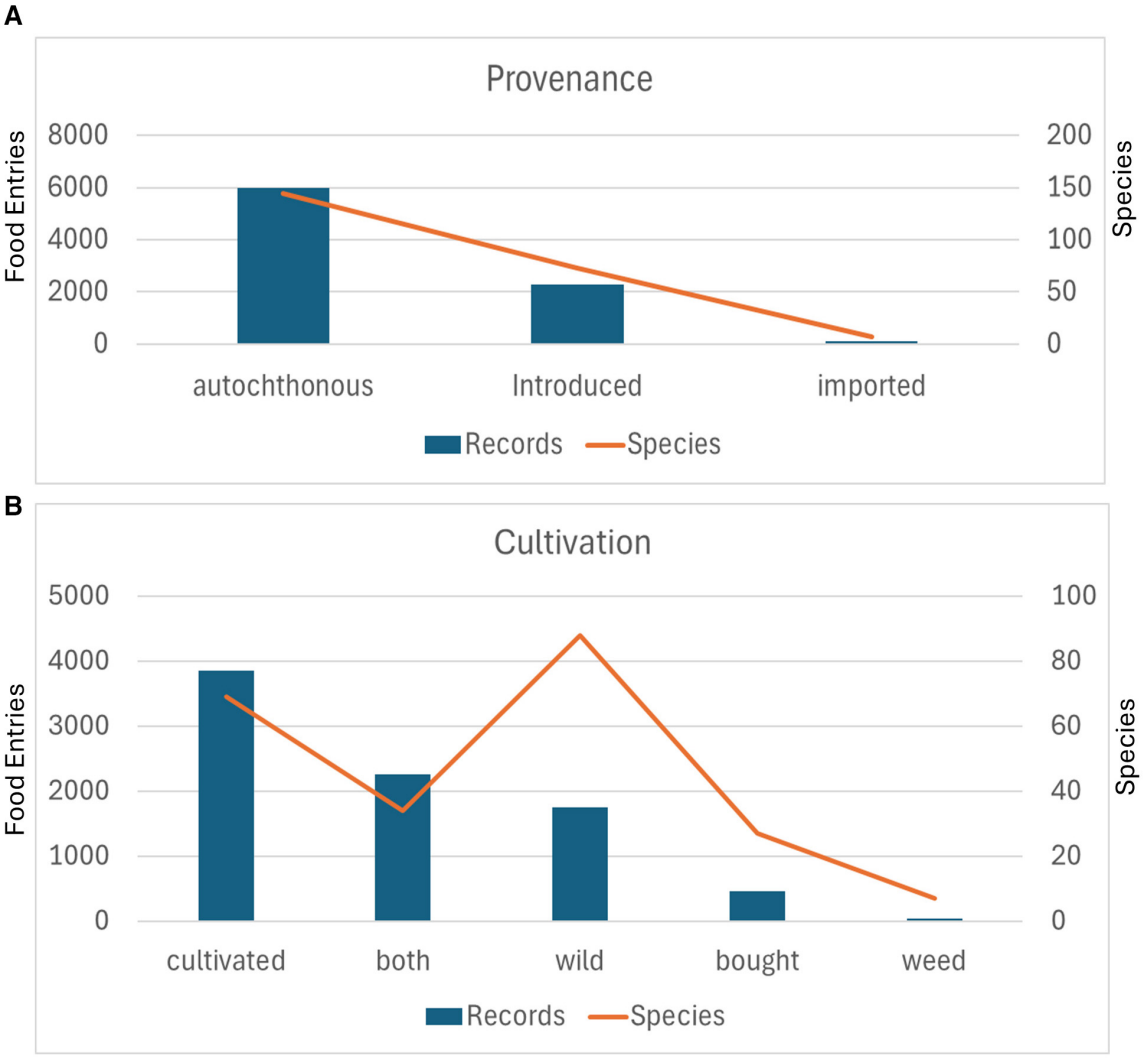
Plant provenance and cultivation patterns in traditional Andean food systems. **(A)** Provenance categories showing autochthonous dominance, with introduced taxa comprising a substantial secondary component. **(B)** Cultivation status revealing balanced distribution between cultivated plants, dual wild-cultivated taxa, and wild species. The inverted relationship between entries and species in the wild category indicates greater species diversity despite lower utilization intensity.

The upper panel of Figure 8 (origin or provenance) demonstrates overwhelming dominance of autochthonous plants (native/indigenous taxa) with approximately 6,000 entries representing 144 species. This constitutes 71% of total entries and 64% of species diversity, reflecting the foundation of Andean food systems on locally evolved biodiversity adapted to highland ecological conditions over millennia. The high species count indicates substantial native plant diversity harnessed through traditional knowledge systems.

Introduced taxa account for ~2,200 entries (73 species), representing approximately 27% of entries and, notably, 32.6% of species diversity. This category includes post-Columbian introductions (Old World crops like wheat, barley, broad beans, and peas) and pre-Columbian exchanges from other South American regions that became integrated into highland agriculture. The higher species-to-record ratio compared to autochthonous plants suggests that introduced species, while larger in number, are utilized less intensively once adopted—even major cereals (*Hordeum vulgare, Triticum aestivum*) and legumes (*Vicia faba*) that complement native pseudocereals.

Imported plants show minimal representation (~100 entries, < 10 species), indicating that the traditional food system relies overwhelmingly on locally established flora rather than continuously imported cultivars. This minimal category represents recently purchased seeds or ornamental/medicinal plants not yet naturalized or those that experience climatic constraints.

The lower panel in Figure 8 (Cultivation and exchange) reveals more nuanced patterns of human-plant relationships across five categories:

Cultivated plants show the highest record frequency (~3,800 entries, 69 species), representing fully domesticated crops under intensive management. Despite highest record counts, this category exhibits moderate species diversity, suggesting intensive utilization of a focused set of staple crops—primarily major tubers (*Solanum* spp.), pseudocereals (*Chenopodium quinoa*), and introduced cereals and pulses.

Dual status (“both”) wild autochthonous plants eventually cultivated (2,252 entries) represent taxa existing in both cultivated and wild forms, often with distinct landraces or varieties under cultivation while wild populations persist. This category is crucial in Andean systems where the boundary between wild and domesticated is fluid—examples include certain *Solanum* species with both wild and cultivated forms, *Oxalis tuberosa*, and various aromatic herbs. The orange line shows a notable dip to 34 species, indicating that this intermediate status applies to a more limited taxonomic set.

Wild plants (1,751 entries) demonstrate an inverted pattern: despite lower record counts than cultivated or dual-status plants, this category shows the highest species diversity. The orange line peaks at 88 species, indicating that many wild species contribute to the food system but individually at lower intensities. This represents gathered plants—seasonal fruits, leafy vegetables, condiments, emergency foods—that supplement staple crops and provide dietary diversity, micronutrients, and resilience during crop failures.

Bought plants (460 entries, 27 species) represent purchased or exchanged items from markets rather than self-produced crops, including commercial varieties of staple crops or specialty items not grown locally.

Weeds show minimal representation (45 entries, 7 species), though even these contribute to the food system—either as tolerated wild vegetables in agricultural fields or as indicator species for land management.

Together, these panels reveal a food system founded on autochthonous biodiversity (native species accounting for 70% of entries) with strategic integration of introduced crops (particularly Old World cereals and legumes). The cultivation gradient from fully domesticated through dual-status to wild demonstrates the hallmark of Andean subsistence: a continuum of human-plant relationships rather than a sharp wild-domesticated dichotomy.

The high wild species diversity (88 taxa) despite moderate record frequency indicates extensive traditional botanical knowledge enabling opportunistic use of diverse resources, while the concentration of entries in cultivated and dual-status categories reflects the reliability requirements of highland agriculture. This pattern—intensive cultivation of proven staples supplemented by extensive gathering from diverse wild sources—provides both productivity and resilience essential for food security in unpredictable Andean environments.

### 3.4 Processing and detoxification

#### 3.4.1 From life forms to detoxification procedures

**Figure 9:**
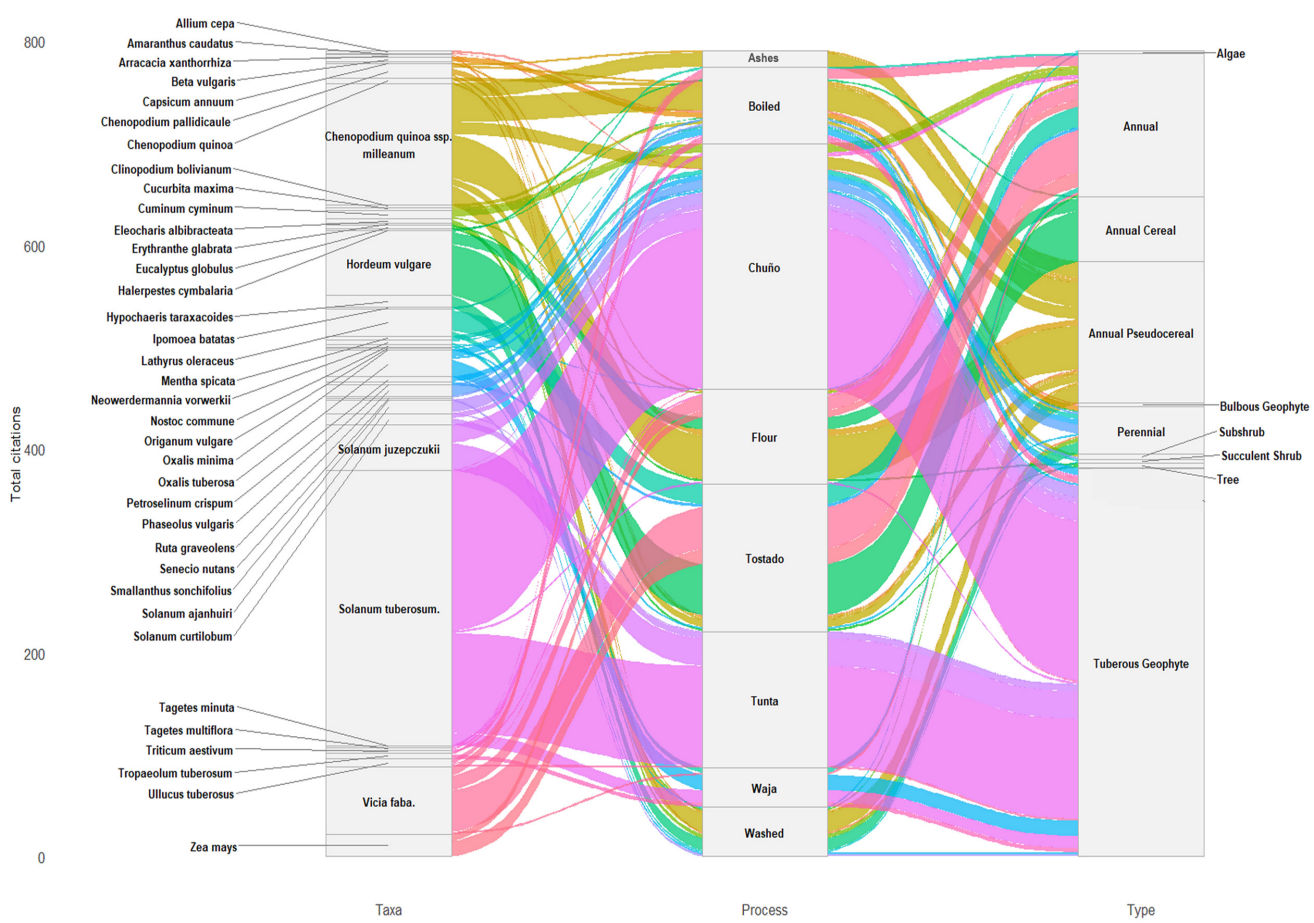
Three-level alluvial plot illustrating relationships among plant taxa, pre-culinary processing categories, and life-form classifications within the dataset. The left axis represents plant taxa (species-level identification), the central axis represents coded pre-culinary processing categories (including preparation and detoxification-related practices), and the right axis represents life-form categories (annual, annual cereal, annual pseudocereal, bulbous geophyte, perennial, subshrub, tuberous geophyte, and tree). Flow ribbons connect categories across axes and represent aggregated counts of coded records (use-reports) linking taxa, processing categories, and life forms. Ribbon widths are scaled proportionally to the logarithm of record frequency to improve visualization of highly skewed distributions. These flows reflect the structure of the coded dataset and do not represent independent observations or statistically comparable quantities. The figure is intended as a descriptive visualization of relationships among coded ethnobotanical variables, rather than as an inferential or evidence-generating analysis. Categories and coding rules are defined in the Methods and Supplementary materials. Figure was created by D.J. Rivera-Obón.

Figure 9 illustrates, in a descriptive and exploratory way, the complex transformation network from taxonomic identities to traditional processing knowledge including critical detoxification protocols that enable consumption of otherwise toxic plant materials and finally botanical life forms. Some taxa show narrow, direct pathways to specific procedures (processing specialization), while others—particularly major staples—exhibit broad fan patterns connecting to multiple procedures (processing versatility). The convergence of multiple colored flows into single procedures demonstrates ingredient diversity and substitutability, while the bifurcation of single taxa toward multiple procedures reveals the processing knowledge maximizing utility from individual crops.

The central panel in Figure 9 aggregates flows into traditional processing categorized by processing method. The Aymara communities of Bolivia have developed sophisticated food processing techniques to detoxify potentially toxic plant materials and render them suitable for human consumption. These methods combine chemical, thermal, and mechanical processes to reduce glycoalkaloid content and other naturally occurring toxins while preserving nutritional value and extending storage life.

Multi-step treatments (freeze-thaw cycles, washing, and cooking) that progressively reduce toxin levels integrate raw material states (fresh, dried, frozen, fermented, and toasted) with transformative techniques (alkaline processing, boiling, kneading, fermentation, freeze-drying, and solar exposure) and culinary endpoints (soups, breads, beverages, snacks, and ritual foods).

Several flows in Figure 9 represent plant taxa, with specific life forms, requiring mandatory processing before consumption. A simple indicator, in our study, of the toxicity inherent in foods and their integration into Aymara culinary practices is, for a specific taxon, the ratio of entries recorded for processes that involve potential detoxification (washing, freezing, roasting, etc.) to the number of entries recorded for that same taxon as an ingredient in various local dishes. Values between 0 and 0.2 correspond to plants generally perceived as safe. Higher values imply cultural significance of the processing. For *Solanum* species, the values range from 0.27 to 1, 0.38 on average, while for *Chenopodium* they are 0.15 on average. This is an explicitly heuristic construct designed to summarize patterns in reported preparation, avoidance, and use across taxa within the dataset. Its purpose is comparative and descriptive, not predictive or mechanistic.

#### 3.4.2 Detoxification pathways through washing for specific food plants

Pseudocereal saponin removal (*Chenopodium* species): The golden/light brown flows come from annual pseudocereals such as quinoa and cañihua, whose seed husks contain bitter saponins, according to the literature discussed below. Traditional processing requires vigorous washing or mechanical abrasion (pearling) before consumption. The flows proceeding to dishes like boiled quinoa, flour preparations, and fermented chicha all presuppose this mandatory detoxification step, though visually represented as direct pathways.

#### 3.4.3 Ash and structural transformation

Ash-Cooking (*Lluxt'a, Llujt'a*; 16 entries, 2 species). This traditional practice, that may warrant further investigation through targeted chemical and functional analyses, involves the controlled combustion of, notably, *Chenopodium quinoa* leaves, stems, and flowers to produce ash that is subsequently kneaded, molded, and dried into a material locally known as *llujt'a*, which is commonly used in conjunction with coca chewing. These observations are presented as ethnobotanical descriptions of reported practices; no chemical or compositional analyses of the ash (e.g., pH, mineral content, or elemental composition) were conducted in this study. While the literature often associates plant ash use with alkaline properties (26), any functional or chemical interpretation remains inferential and is not evaluated here. Compacted ash of *cañihua* (*Chenopodium pallidicaule*) in pieces 2.6 cm thick, 6.9 cm in maximum diameter is reported as used by Quechua communities of Puno (Peru) as a catalyst for coca chewing (27).

#### 3.4.4 Cryogenic preservation systems

**Figure 10:**
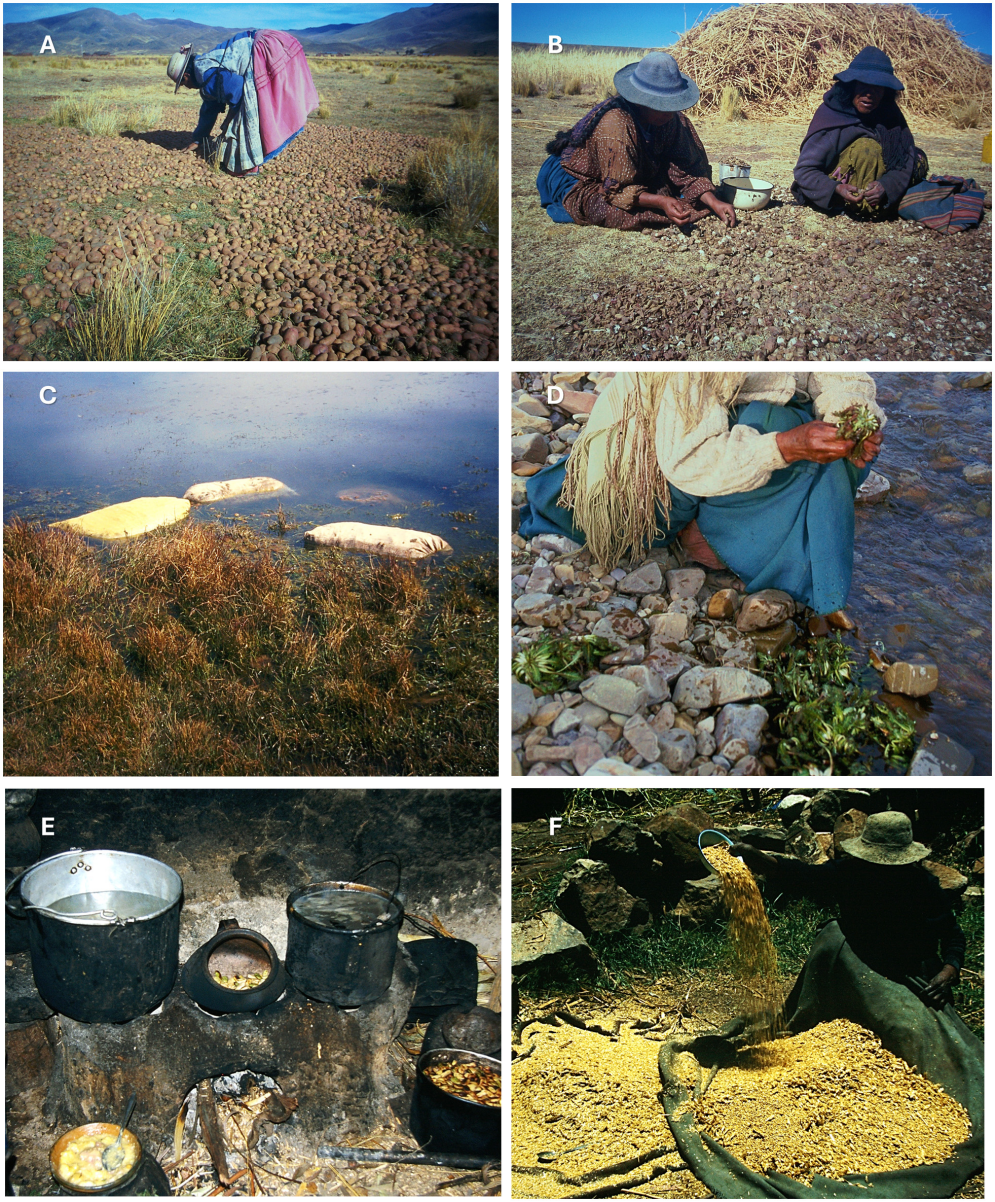
Traditional Aymara food processing and detoxification methods for plant-based foods in Bolivia. **(A)** *Chuño* production, exposing potatoes to freeze-drying cycles by arranging them on the ground under night frost and daytime sun. **(B)** Peeling and drying of *chuño*. **(C)** *Tunta* preparation, submerging pre-processed potatoes in sacks within a lake for further treatment. **(D)** Washing *Hypochaeris meyeniana* (*pilli/jallu sik'i*) for raw consumption. **(E)** Cooking and toasting using a traditional Puni hearth. **(F)** Initial step (winnowing) in the processing of *Hordeum vulgare* for the production of pearled barley.

Andean freeze-drying (Figure 10) exploits the extreme diurnal temperature swings of the altiplano—sub-freezing nights followed by intense solar radiation—to produce shelf-stable tuber products from potato (*Solanum* spp.), oca (*Oxalis tuberosa*), and isaño (*Tropaeolum tuberosum*). The technique serves two simultaneous purposes: long-term preservation and detoxification, since the repeated freeze-thaw cycles combined with water immersion progressively leach the glycoalkaloids that make bitter potato varieties inedible in their raw state. All freeze-dried products require rehydration before final preparation, typically by boiling, steaming (*phuti*), or incorporation into soups. Further details are available at Supplementary Table 1.

Three main products are documented, differing in processing intensity and end use. *Ch‘uño* (black *chuño*) is the least processed variant, relying on natural frost exposure, trampling, and sun-drying without water immersion; it retains some residual bitterness but can be stored for years (252 entries, 26 species, notably *Solanum* spp.). *Tunta* (white *chuño*; 134 entries, 10 species) undergoes the most intensive treatment—multiple freeze-thaw cycles followed by 3–4 weeks of submersion in a purpose-built water reservoir *(tunta qüña*) fed by running water (Figure 10)—yielding a near-white, maximally detoxified product suited to commercial sale and multi-decade storage. A household variant submerged in still rather than running water produces a darker, lead-gray product. *Kaya* applies an analogous process to oca (17 entries), with two variants distinguished by whether water immersion precedes (*uma kaya*) or follows (*juyphi kaya*) the freezing stage. Among preparations derived from these products, *tunta phuti* is preferred over *ch'uño phuti*, and *uma kay phuti* is *preferred* over *juyphi kay phuti*. All three base products can also be incorporated into soups, with specific preparations for chopped and flour forms. When stored in cool, dry conditions, all three keep for 5 years or more, functioning as a form of inter-annual food security against harvest failures.

This climate-adapted technology provides extreme shelf-life (multi-year storage), produces lightweight products facilitating transport and trade, and transforms the temporal dimension of food security by converting seasonal harvests into year-round staples. The cultural logic positions processing as temporally primary over cooking, with food functioning as insurance against harvest failures and enabling inter-annual surplus management.

#### 3.4.5 Thermally processed grain and legume

Several complementary heat-processing techniques form the backbone of Aymara food preparation, each serving distinct nutritional, practical, and social purposes.

Dry-heat toasting (*jamp'iña*; *tostado*) is the dominant pathway for cereals, legumes, and pseudocereals (146 entries, 13 taxa). Grains are toasted whole for immediate consumption or ground into flour (*aku/pitu*), which serves as a versatile base for porridges, soups, beverages, and breads. Thermal processing reduces antinutrients—particularly lectins and protease inhibitors in legumes—while enhancing digestibility through protein denaturation and starch gelatinization. For elderly individuals with limited dentition, aku often constitutes the primary source of nourishment. Dry-roasting followed by grinding is the standard sequence for a wide range of seeds including broad bean, quinoa, cañihua, barley, and maize.

Boiling (*qhatiyaña*) is applied to tubers and whole grains (77 entries, 20 species). Boiled tubers (*qhati*) accompany soups at morning and evening meals or are carried as packed midday meals (*ququ*) during agricultural work. Whole-grain preparations (*mut'i*) require extended cooking and are typically set in the evening to complete overnight. A variant (*allintat mut'i*) pre-roasts the grains to shorten cooking time. In Aymara food culture, the main meals consist of a soup paired with a solid or “dry” preparation (*waña manq'a*)—a term used in the strictly literal sense to distinguish solid food from liquid.

Pit-roasted stew (*waja*) is a communal technique combining roasting and steaming in a sealed earth oven (38 entries, 8 species). The oven is built from soil clods or stone, heated until red-hot, then collapsed over the food and sealed to retain heat. It is typically prepared on harvest days or following ritual sacrifices (*khuchu*).

Frying (*kaswira/thixi*) using oil or pork fat is applied to meats, eggs, tortillas, and fritters. Two further methods address barley hull removal: *thijuta*, an older technique of toasting and hand-rubbing grains in sheepskin, now largely obsolete; and *phata*, in which soaked barley is hand-pounded and washed to yield pearled grain for soups or stews.

Further details are available at Supplementary Table 2.

#### 3.4.6 Controlled microbial transformation

The aim in this section is to record and systematize reported practices, terminologies, and process logics as described by participants—not to reproduce a food-process engineering or microbiological study. Therefore, no measurements of pH, water activity, microbial communities, or contamination were conducted, and the analysis is limited to reported practices and their comparative structure. Deliberate decay and fermentation are applied to potatoes, legumes, and quinoa through processes such as soaking, partial decomposition, and microbial fermentation prior to final thermal treatment (e.g., roasting or boiling). Products such as *phusphu* (from broad beans and peas; 27 entries, 3 species), *k'usa* (from quinoa), and *muray phuti* (from potato) likely result from controlled microbial activity that enhances flavor, modifies nutritional properties through partial pre-digestion, and generates foods with ritual and cultural significance. Within this framework, decay is not construed as spoilage but as a productive transformation, challenging conventional distinctions between fresh and spoiled foods. Here we do not establish safety thresholds or process controls in a technical sense, and no claims are made regarding microbiological safety in the absence of direct analysis Microbiologists actively analyzed the microorganisms involved in these fermentation processes, but they concentrate specially on *K'usa* and other beverages (28, 29) therefore it is important to draw their attention to other under investigated fermentations here described.

*Phusphu* consists of dried seeds of broad bean (*Vicia faba*) or pea (*Pisum sativum*), which are soaked and subsequently roasted—sometimes after parboiling—in a traditional toaster (*jiwk'i*), yielding a softer and more portable food. *K'usa* is a fermented beverage traditionally prepared by chewing quinoa or maize flour to initiate fermentation; although formerly consumed during festivals, it has largely been replaced by commercial alcoholic drinks. *Muraya* refers to potato tubers that develop a fermented odor following prolonged submersion in water, either intentional or incidental; when steam-cooked, the product is known as *muray phuti*.

#### 3.4.7 Direct consumption with minimal processing

Certain plant materials undergo minimal or no processing beyond mechanical mastication, boiling, or sucking juices, particularly fresh green beans, tender maize stems, and flowers consumed during agricultural labor (903 entries and 102 species). These items provide seasonal nutrition, immediate energy during fieldwork, and represent food consumption patterns embedded within labor activities rather than constituting formal meals. The cultural logic distinguishes between sustenance and meal events, recognizing snacking and ritual tasting as legitimate food practices outside structured dining contexts.

Flour (*aku* in barley, *aku* and *aqallapu* in quinoa): Milled products (93 entries, 12 species) are produced from previously detoxified grains and tubers. Ground preparations from tubers include dried *muraya, tunta*, or *chuño*. Toasted ground grains include *pitu* and *aku*.

*Allintata* (190 entries) is a detoxification process applied to grains, involving dry-roasting in a *jiwk'i* (traditional clay roaster) followed by boiling until fully cooked, yielding, from corn, *allintat tunqu* or *tunqu mut'i*. The process is also applied to broad beans and peas, producing *allintat jawasa* and *allintat alwirja*, respectively.

### 3.5 Diversity of uses

**Figure 11:**
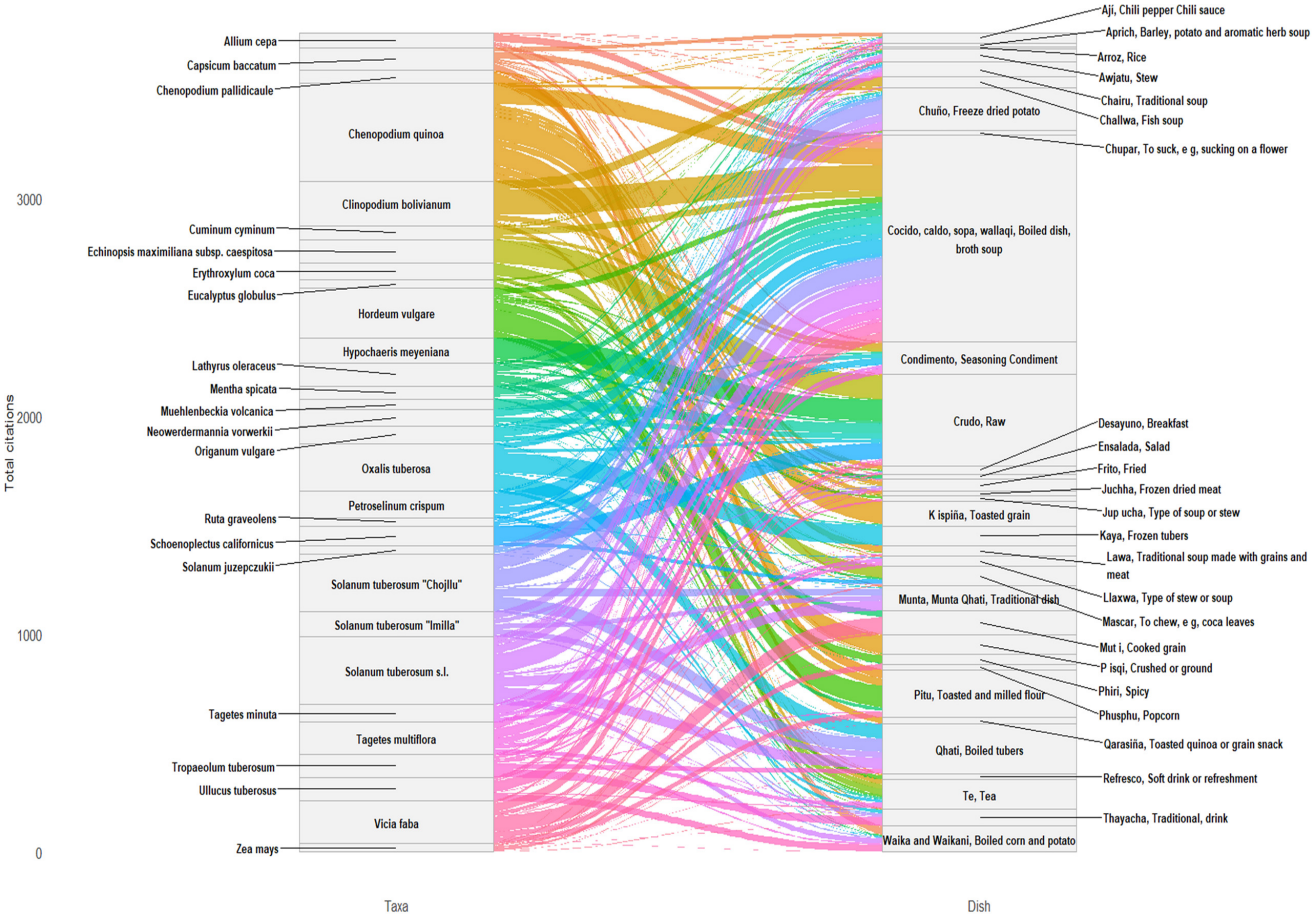
Alluvial plot illustrating the relationships between the 30 edible plant taxa most frequently cited by informants **(left)** and the traditional Andean food preparations **(right)** in which they are used. The width of each flow is proportional to the number of recorded associations between a given plant species and specific food products. Colors differentiate individual plant taxa and trace their pathways through the traditional food system. The flows do not represent strictly comparable quantitative units, but rather structured transitions between coded dimensions of the dataset. Figure was created by D.J. Rivera-Obón.

The plant-to-food transformation is described and explored in Figure 11 that reveals a strongly skewed distribution: a small number of broad preparation categories account for the majority of entries, while a rich array of culturally specific dishes each contributes at lower frequency. The categories used in this analysis are not drawn from a formal or external classification system. Instead, they emerge directly from informants' repertory, mirroring how foods, preparations, and consumption practices are locally named and understood. The dataset intentionally retains this emic perspective, avoiding reorganization into a standardized or hierarchical taxonomy.

The apparent heterogeneity—spanning ingredients, processing states, named dishes, and consumption contexts—reflects the structure of local knowledge systems, where such distinctions are not always analytically separated. To ensure cultural and linguistic consistency, the classification was reviewed and interpreted by an Aymara researcher on our team (SCY).

Major staple crops—quinoa, potato, maize, and broad bean—show the greatest processing versatility, each feeding into five or more distinct preparations, while minor crops and wild plants connect to narrower, more specialized pathways. A middle convergence zone in the diagram reflects recipe complexity and ingredient substitutability: preparations such as cocido, various chuño dishes, and fermented beverages draw from multiple botanical sources simultaneously.

Soups and broths constitute the dominant preparation mode, with dishes such as cocido, caldo, sopa, and *wallaqi* collectively numbering 1,200 entries and 64 species. Boiling thus emerges as the primary vehicle for incorporating plant ingredients into daily meals. Raw consumption ranks second (903 entries, 102 species), suggesting a significant role for unprocessed plants as snacks, ritual foods, or accompaniments. Infusions (mate/té, 439 entries, 73 species) form a third major domain, indicating that liquid nourishment—both as meals and beverages—is central to Aymara plant use.

Processing specialization is evident in certain pathways. Some plants contribute to single, highly specific traditional foods, shown by narrow, direct flows, while others (particularly the major staples) exhibit broad, fan-like distributions connecting to 5–10 or more different food products. This reflects both crop versatility and the depth of traditional processing knowledge.

The middle convergence zone reveals that certain traditional preparations draw from multiple plant sources (visible where flows of distinct colors converge), indicating recipe complexity and ingredient substitutability in traditional cuisine. Products like “*Cocido*” (stew), various *chuño* preparations, and fermented beverages aggregate flows from diverse botanical sources.

**Figure 12:**
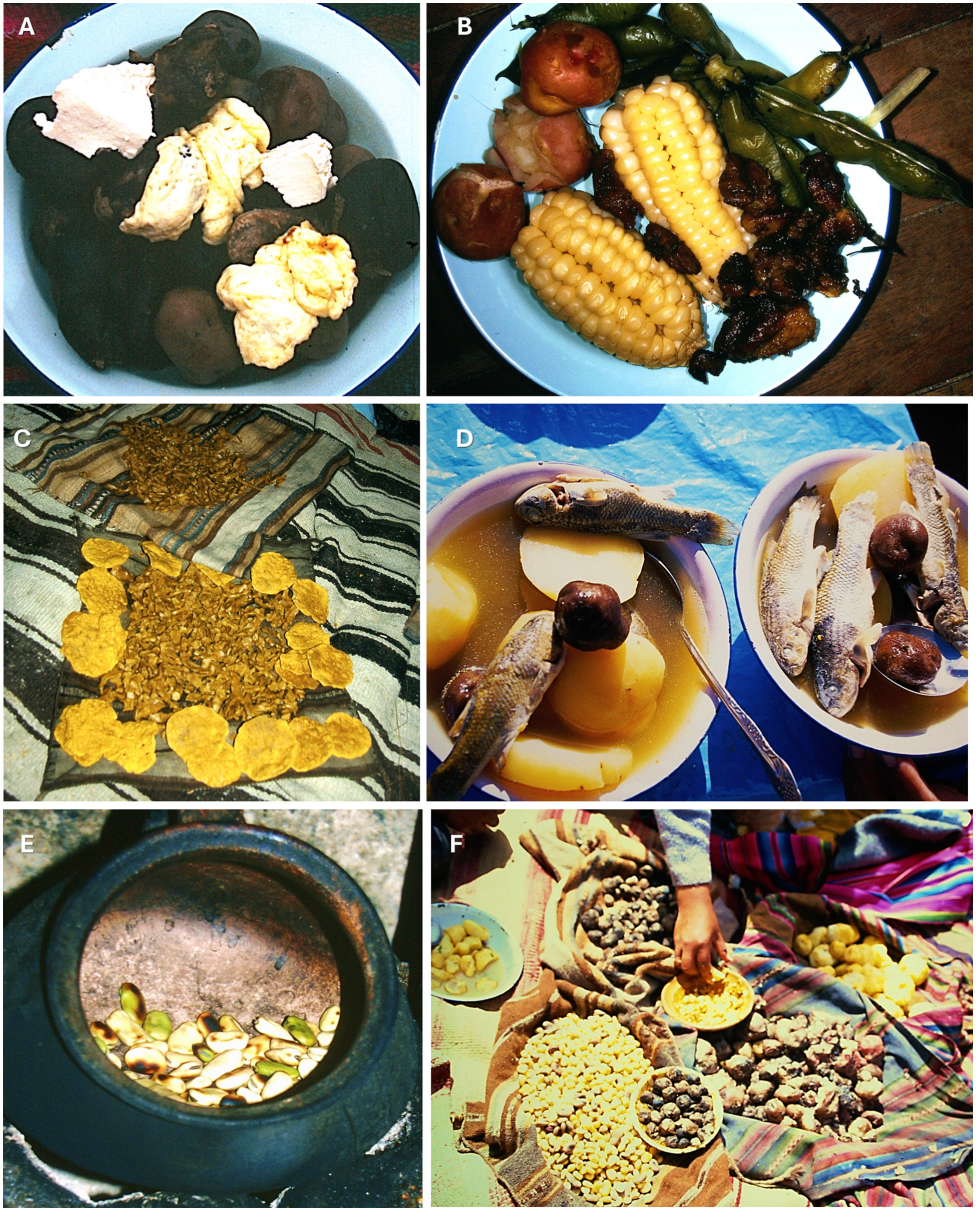
Traditional Aymara Bolivian dishes from the highlands. **(A)** *Chuño negro* served with cheese. **(B)** *Plato paceño*, a typical dish from La Paz. It typically includes a combination of boiled corn (*choclo*), boiled green beans (*habas*), boiled potatoes, and often also features other ingredients. **(C)** *Qarasiña* and *k'ispiña*, both prepared using quinoa as the primary ingredient. *K'ispiña* (center) and *qarasiña* (round cakes). **(D)** *Wallqi* or *chalwa kaltu* (fish soup), which is made from potato, *chuño* (freeze-dried potato), and *Orestias luteus* Valenciennes, 1846 (a species of fish from the lake). **(E)** Toasted broad beans (*habas*) prepared in a *jiwki* (clay hearth). **(F)** Highland snack (*merienda*) in Puni, featuring corn, chuño, potatoes, and *katachi* soup.

Beyond these dominant categories, a specialized repertoire of named dishes is documented, each with moderate to low citation frequency. These range from staple flour preparations (*pitu, phiri*) and freeze-dried potato dishes (*ch'uñu kaltu, tunta phuti*) to saponin-neutralized quinoa products (*k'ispiña, qarasiña, p'isqhi*; Figure 12), condiments (*wayk'a*), and cold-weather preparations (*thayacha, jup'ucha*). The dataset also reflects the bilingual nature of Aymara culinary culture: Spanish-language terms such as *ensalada, arroz*, and *frito* sit alongside Aymara names, pointing to the ongoing integration of mestizo dishes. Notably, coca mastication (masticar, 141 entries) is classified as a form of food intake rather than purely a ritual or stimulant practice.

Traditional Aymara cuisine constitutes a sophisticated plant-based food system in which nutritional strategy and cultural identity are deeply intertwined. The dominance of boiled preparations—soups and broths such as *wallaqi, qhati*, and *cocido*—reflects an adaptive response to high-altitude Andean conditions, maximizing nutrient extraction from local flora, while raw consumption and infusions broaden the dietary repertoire across both solid and liquid forms. The rich lexicon of specialized dishes, from *ch‘uñu kaltu* and *p'isqhi* to *k‘ispiña* and *mut'i*, encodes generations of culinary knowledge tied to specific ingredients, techniques, and social contexts. The coexistence of indigenous and Spanish culinary terminology signals a living, adaptive gastronomic tradition, and the formal inclusion of coca mastication underscores the inseparability of nutrition and ritual in Aymara foodways. Spanning primarily 30 plant taxa and 34 distinct preparations, but involving up to 227 taxa, this cuisine stands as a nutritionally diverse, ecologically grounded, and culturally expressive system central to Aymara community life.

## 4 Discussion

### 4.1 Biocultural significance and knowledge transmission

In this study, we have observed the high value placed on food as an integral part of community life through rituals and collective celebrations, with certain dishes reserved specifically for these occasions. Furthermore, it is common for daily meals to be eaten in the fields, during agricultural work or herding, for which special dishes are prepared that are easy to transport and share. This communal aspect has been very significant in the daily life of the Aymara (11). Van Kessel (30) argues that Andean agro-pastoral technology operates within two interrelated dimensions: the technical-empirical and the religious-symbolic. The latter organizes plants through production rituals that structure the rational exploitation of high-Andean resources (30). This ethnobotanical classification system mobilizes ecological knowledge—encompassing climatic variations, soil properties, and plant physiology—to support both wealth generation and cultural reproduction (30).

We detected a higher taxonomic resolution among Aymara informants with respect to the botanical and agronomic taxonomies, concerning local crops, notably *Solanum* and *Chenopodium* species. Critically, Aymara taxonomy provides higher “taxonomic resolution” at the varietal level than Linnaean classification, a distinction essential for managing biodiversity in the Titicaca basin (31–33). This should explain the numerous *Solanum* sub-categories documented in our dataset that are absent from standard botanical references (18, 21, 34). Unlike Western nomenclature, Aymara plant names encode environmental or functional descriptors rather than morphological characters (35) and exhibit “one-to-many” or “many-to-one” relationships with Western species concepts (36). Plants may be grouped by criteria such as cooking time or processing requirements—biological realities overlooked by conventional taxonomy but critical for nutritional science (37). The preservation of Aymara taxonomic systems is therefore essential not only for cultural continuity but also for the conservation of Neotropical food genetic diversity (37).

The relationship between Aymara cosmovision, environmental knowledge organization, and subsistence practices—cultivation, harvesting, detoxification, processing, storage, and consumption—is particularly evident in collective social contexts such as community festivals. Each element within this integrated system occupies a precise functional role and is designated by highly detailed nomenclature. Consequently, the preservation of food names and processing terminology constitutes a critical mechanism for safeguarding traditional techniques that are otherwise at risk of erosion.

Our findings reveal specialized food knowledge concentrated among women and marked intergenerational differences in knowledge transmission following a bimodal distribution. Bimodal patterns in social systems have been documented in diverse contexts, including economic cycles (38), human communication networks, trading behavior, and age-stratified tourism patterns (39, 40), and are typically interpreted as evidence of regime-switching dynamics or “social bubbles” (38). In the present case, the bimodal structure may reflect competing epistemological regimes: one rooted in traditional ecological knowledge, the other oriented toward external socio-economic integration.

Our findings reveal specialized food knowledge concentrated among women and marked intergenerational differences in knowledge transmission following a bimodal distribution. This pattern may reflect the coexistence of traditional ecological knowledge systems and externally oriented socio-economic frameworks, as observed in other indigenous contexts (41). The apparent decline in knowledge transmission among younger cohorts raises concerns regarding the continuity of ethnobotanical practices.

From an applied perspective, these results highlight the need for targeted biocultural conservation strategies. These may include: (i) integrating Aymara ethnobotanical knowledge into formal and informal education systems; (ii) supporting community-led documentation of plant nomenclature and processing techniques; and (iii) strengthening the role of women as key knowledge holders in agrobiodiversity management. Such approaches are essential not only for cultural preservation but also for maintaining locally adapted food systems that contribute to resilience under conditions of climatic and socio-economic change.

### 4.2 Nutritional potential of local techniques

In this and the next section all external nutritional or bioactivity literature is cited strictly as contextual background supporting plausibility, not as empirical validation of outcomes in the present dataset. From an ethnobotanical perspective, this work documents reported uses and locally described handling and preparation practices for these taxa, without validating their nutritional or toxicological profiles. While external literature suggests possible nutritional benefits or contaminant risks under certain conditions, such interpretations are not empirically verified here.

Potato species (*Solanum* sp.pl.) are prevalent as food items among Aymara communities (15 taxa, 857 local dish entries, 339 processing entries). Several studies (32–46), consistently demonstrate the high nutritional value and remarkable diversity of native Andean potatoes, highlighting their importance for food security, dietary quality, and biodiversity conservation. Native Andean potato landraces exhibit substantial variability in nutritional composition, including protein, iron, zinc, calcium, antioxidant capacity, and phenolic content. Beyond compositional richness, varietal diversity plays a functional dietary role in high-altitude Andean food systems, where native landraces contribute to energy, protein, iron, and zinc intake, particularly among women and children, and help buffer seasonal food shortages. These attributes underscore the nutritional significance of Andean *Solanum* diversity, especially for indigenous and economically vulnerable populations (32–46). The findings of Yábar-Villanueva et al. and Torres-Gutierrez et al. underscore how the proper selection of potato variety and freezing temperature can optimize the nutritional, functional, and sensory characteristics of *chuño* (47, 48).

The 339 potato processing entries in our study refer likely to detoxification. Glycoalkaloids are secondary plant metabolites that at appropriate levels may be toxic to bacteria, fungi, viruses, insects, animals, and humans. Potato glycoalkaloids may have evolved in nature to protect the plant against phytopathogens and other hostile environments. The potential human toxicity of glycoalkaloids has led to the establishment of guidelines limiting the glycoalkaloid content of new cultivars (49). *Chuño* and especially *Tunta* processing contributes to the reduction of alkaloids and, in parallel, of phenolics and micronutrients (50–52). But also improve their nutritional profile by increasing their GABA and resistant starch content (53, 54).

*Chenopodium* species follow potatoes in order of relevant food items among Aymara communities (5 taxa, 536 local dish entries, 138 processing entries). Empirical toxicity index values range from 0.13 to 1.7, mean 0.15, exception made of *Chenopodium quinoa* var. *melanospermum* where it reaches 1 but with low support. *Chenopodium quinoa* and *Chenopodium pallidicaule* are nutritionally exceptional pseudocereals, surpassing conventional cereals in protein quality (13%−18%, balanced essential amino acid profile with relatively high lysine content and high digestibility), lipid content (5%−9%, predominantly unsaturated fatty acids with tocopherols and minor amounts of squalene), contain minerals such as calcium, iron, and magnesium, although bioavailability is influenced by phytate content and processing, B-complex vitamins, and dietary fiber (7%−10%) (55). Both are naturally gluten-free and contain bioactive compounds with well supported antioxidant properties (56, 57). Some authors support their role as functional foods in the prevention of chronic metabolic diseases (56, 57). Their capacity to address lysine deficiency and low mineral bioavailability in monocereal-based diets is reported by (58) of particular relevance for Andean populations, though nutritional composition varies across cultivars and processing methods. Traditional detoxification of *Chenopodium quinoa* seeds—typically involving abrasion, washing and, in some protocols, pearling to remove bitter saponins—substantially reduces saponin content and improves palatability and digestibility, but can also lead to losses of minerals, phenolic compounds and other nutrients depending on the intensity and method of processing, underscoring the need to optimize treatment conditions to maximize nutrient retention while achieving effective antinutrient reduction (e.g., mineral losses with polishing and boiling vs. higher retention with steaming) (59).

*Ullucus tuberosus* (*ulluco*) presents 104 local dish entries, but only 8 processing entries, with a low toxicity index value of 0.08. According to Ref. (60) it features brightly colored tubers with 80%−87% moisture, 1%−2.5% protein, and 10%−15% carbohydrates (primarily starch), providing 40–60 kcal/100 g. While not protein-rich, it contributes carbohydrates and micronutrients to highland diets and has traditional medicinal uses.

*Tropaeolum tuberosum* (*isañu/mashua*) presents 104 local dish entries, but only 6 processing entries, with a low toxicity index value of 0.06. In addition to the tubers, the leaves are also used. It provides (61–63) dietary fiber, potassium, phosphorus, vitamin C, and modest protein. Pigmented genotypes are rich in phenolic compounds (flavonoids, anthocyanins) with strong *in vitro* antioxidant capacity.

*Oxalis tuberosa* (*oca*) with 219 local dish entries, but only 26 processing entries, presents a low toxicity index value of 0.12. According to Ref. (64, 65) it exhibits diverse morphology and color (white to deep purple) and is classified as sweet or sour, reflecting taste, oxalate content, and processing. It contains flavonoids, cinnamic acid derivatives, and anthocyanins, contributing to antioxidant potential. High oxalate levels may reduce calcium bioavailability, but traditional sun exposure and dehydration (e.g., *juyphi khaya, uma khaya*) mitigate this and enhance sweetness, supporting food security (64, 65).

*Arracacia xanthorrhiza* (*arracacha*; with only 3 local dish entries, presents a high empirical perceived toxicity index value of 0.7), is the nearly sole New World Apiaceae domesticated for its storage root. According to (66, 67) it is valued for its digestibility, small starch granules, mineral content, and phenolic compounds like chlorogenic acid. Nutritionally comparable to other starchy roots, its culinary versatility (soups, stews, breads, and beverages) and post-harvest susceptibility influence its cultural and economic importance. However, EFSA (European Food Safety Agency) raised concerns on the viability of *Arracacia* as a novel food in Europe because of the lack of data in the proposal concerning, among others, data on the presence of toxic secondary metabolites, in particular of coumarin and monoterpene derivatives (68). This coincides with the implicitly perceived toxicity of this root among the Aymara.

Interest in *Lupinus mutabilis* (*tarwi*) seeds, stems from its protein, essential fatty acids, minerals, and fiber, promising against malnutrition but constrained by bitter/toxic alkaloids varying by environment (69). Nevertheless, it was rarely mentioned by our informants (only four instances as an ingredient in local dishes). Despite the well-known toxicity of *Lupinus* seeds (69), we did not record any processing methods for this species, possibly due to its apparent lack of cultural significance.

The documented technological repertoire reveals empirical practices related to detoxification, freeze-drying, thermal processing, and fermentation that appear to have developed through long-term local experimentation. In many cases, processing complexity exceeds culinary preparation, indicating that the transformation of raw materials constitutes a central domain of culinary expertise within Andean food systems.

Traditional processing strategies—such as *chuño* and *tunta* production or quinoa desaponification—represent locally refined techniques that could inform context-specific nutrition intervention studies aimed at enhancing the bioavailability, safety, or functional characteristics of regional foods, particularly under high-altitude and resource-limited conditions. Similarly, the ethnobotanically recorded diversity of potato and quinoa landraces underscores the potential of agrobiodiversity-based food systems to support dietary diversity and resilience to climatic variability.

### 4.3 Nutritional potential of lesser known sources of food

The Aymara communities studied consume the bases of totora stems, generally eating them raw (73 instances) if they are tender enough, or simply chewing them (17 instances). Edible basal stems, rhizomes, and shoots of *Schoenoplectus californicus* (totora), traditionally consumed in lakeshore communities including Aymara around Lake Titicaca, provide moderate levels of protein (≈7%−10%), high dietary fiber, carbohydrates and substantial minerals such as calcium, iron, potassium and exceptionally high iodine, indicating potential as a non-conventional nutrient source, although the species' ability to accumulate environmental contaminants (e.g., heavy metals) when grown in polluted wetland systems underscores the importance of environmental assessment and monitoring to mitigate potential toxicity risks before consumption according to Loza-Del Carpio et al. (70). Girault lists the medicinal uses of the plant's stems and leaves, both fresh and dried, some of which were already recorded by chroniclers from the 16th century onwards (71).

*Nostoc commune*, with 34 entries as an ingredient in local foods, is of some significance; it is consumed cooked or in traditional stews and only occasionally raw (eight entries). Only in two cases were we told that it needed to be washed beforehand, although this may have been implied in other cases. Our entries of *Nostoc* intake come from the provinces of Camacho and Los Andes. Several studies prove that *Nostoc commune* is not just a “filler” food. It contains significant amounts of calcium, iron, and dietary fiber, which allows to argue that for Aymara communities with limited access to dairy*, Nostoc (llayta* or *murmunta)* serves as a critical traditional source of calcium for bone health (72, 73). However, Johnson et al. (74) warned on the presence of a neurotoxic amino acid in *Nostoc commune* consumed in the Peruvian highlands.

The potential risks posed by environmental contaminants (e.g., in *totora*) and naturally occurring compounds (e.g., in *Nostoc commune*) underscore the importance of integrating food safety considerations into discussions of these species. However, this study did not conduct site-specific measurements of heavy metals, toxins, or other contaminants; thus, any assessment of actual risk levels remains beyond the scope of the present data.

This documentation does not constitute evidence of dietary adequacy, safety, or suitability for public health or nutritional programming. Instead, it highlights the need for interdisciplinary research—encompassing environmental monitoring, phytochemical characterization, and food safety assessment—to evaluate the potential roles of these species in food systems.

Finally, the inclusion of these taxa in ethnobotanical inventories contributes to broader efforts in documenting biocultural diversity and identifying candidates for future research, rather than serving as a basis for immediate nutritional or policy recommendations.

### 4.4 Overall implications for policy and practice

Taken together, the results document a structured Aymara food system characterized by the integration of agrobiodiversity, traditional food processing practices, and culturally embedded knowledge systems. The findings provide a descriptive ethnobotanical account of plant–food relationships and associated practices, and do not constitute evidence of nutritional outcomes, dietary adequacy, or food system performance.

Within nutrition-sensitive food systems approaches, such as those increasingly emphasized in global frameworks on food biodiversity and sustainable diets, descriptive documentation of local food resources represents an important upstream contribution to understanding how dietary diversity is structured in practice. In this sense, the present study contributes to the characterization of food system diversity and traditional knowledge relevant to food use, rather than to the assessment of nutritional status or health effects.

The references to resilience, sustainability, and food sovereignty are used as interpretive framing concepts consistent with their application in food systems research and nutrition ecology. These concepts are widely employed to contextualize how local food knowledge systems intersect with broader sustainability transitions and biodiversity conservation agendas, including those aligned with global development priorities such as sustainable food systems and the conservation of agrobiodiversity. However, these frameworks are not operationalized or empirically tested in this study.

It is explicitly acknowledged that the dataset does not include measurements of household vulnerability, food access, affordability, dietary intake, or nutritional biomarkers. Consequently, no intervention, policy prescription, or programmatic recommendation is derived from the results. However, from a nutrition systems perspective, structured documentation of locally used plant resources and processing knowledge provides a necessary empirical foundation for subsequent stages of research in the “evidence pipeline,” including food composition analysis, dietary assessment, and nutrition-sensitive intervention design.

Accordingly, the findings should be interpreted as a baseline contribution to the documentation of food biodiversity and ethnonutritional knowledge systems. Such baselines are increasingly recognized as essential for identifying underutilized crops and traditional food practices that may warrant further investigation within interdisciplinary efforts linking nutrition, agriculture, and biodiversity conservation, including those aligned with global priorities on sustainable diets and resilient food systems.

## 5 Conclusion

This study documents ethnobotanical knowledge embedded in Aymara food systems through systematic analysis of plant–food associations and reported processing practices. The results describe how plant resources, culinary transformations, and use contexts are organized within a culturally structured knowledge system. The alluvial representations are exploratory visual tools designed to illustrate relationships among coded ethnobotanical categories. They are intended to support descriptive analysis of knowledge structure and diversity rather than to establish causal, nutritional, or functional relationships.

A total of 213 plant species and subspecies were recorded, reflecting documented use and knowledge within the study population. These records represent ethnobotanical observations and should not be interpreted as evidence of nutritional efficacy, safety, or physiological effects. Any such interpretations require dedicated compositional, toxicological, and dietary assessment studies.

Traditional processing practices—including freeze-drying (*chuño, tunta, kaya*), soaking, fermentation-like transformations, and other culinary techniques—are described as reported methods for rendering plant materials edible and storable under high-altitude environmental conditions. While the broader literature on underutilized crops and traditional food systems has highlighted potential biochemical and nutritional relevance of such practices, no compositional or functional analyses were conducted in this study.

The documentation of Aymara food nomenclature and classification systems highlights a structured ethnolinguistic organization of food knowledge, contributing to broader efforts in the documentation of food biodiversity and traditional knowledge systems. Such documentation is increasingly recognized as relevant for understanding the diversity of food systems and for supporting future research on underutilized crops and culturally embedded dietary practices.

In conclusion, this work provides a systematic ethnobotanical baseline of Aymara food systems. Its primary contribution lies in documenting and structuring plant–food knowledge in a way that can inform future nutrition-sensitive research, particularly studies integrating food composition analysis, dietary assessment, and sustainability-oriented food system evaluation in Andean and other high-biodiversity agroecological contexts.

## Data Availability

The datasets presented in this study can be found in online repositories. The names of the repository/repositories and accession number(s) can be found below: AymaraFoodGit_16_4_26.xlsx at https://github.com/drivera2001/Aymara-Food.
