# Supplementary material for: From tradition to nutrition: wild and cultivated Aymara food plants as sustainable resources in the Andean neotropics of Bolivia
Source: Front Nutr. 2026 May 22;13:1826362. doi: 10.3389/fnut.2026.1826362 (PMC13238266; doi:10.3389/fnut.2026.1826362)
Supplement: Supplementary file 1 [file Table_1.docx]

## Supplementary Tables

### Supplementary Table. T. 1. Cryogenic Preservation Systems

| Product | Aymara term | Base ingredient | Processing steps | Final preparation | Use / notes |
| --- | --- | --- | --- | --- | --- |
| Ch'uño | *ch'uñu / juyphimp pasayaña* | Bitter potato (*Solanum* spp.) — 10 species/subspecies; also some sweet varieties (e.g. *saq'ampaya*) | Spread to freeze overnight → trampled to expel moisture → skin removed → sun-dried. No water immersion. | ch'uñu phuti (steam-cooked); chayru (soup with chopped ch'uñu); ch'uñu allp kaltu (soup from ch'uñu flour) | Retains some residual bitterness. Stores for years. 241 records, 23 species. Some informants linked the process also to Oxalis tuberosa and quinoa detoxification. |
| Tunta | *tunta / muraya (white chuño)* | Bitter potato (Solanum spp.) — 10 species | Spread to freeze 2–5 nights (shielded from sun during day) → submerged in running water in tunta qüña (purpose-built reservoir) for 3–4 weeks → re-frozen → trampled to remove skin → sun-dried. | tunta phuti (soaked, pressed, steam-cooked); also used in soups | Near-white colour; maximum glycoalkaloid removal. Designed primarily for commercial sale. Stores for decades. 134 records, 10 species. Preferred over *ch'uñu phuti.* |
| Tunta (household variant) | *tunta (darker variant)* | Bitter potato (Solanum spp.) | Submerged in still water for 3–4 weeks → frozen → trampled to remove skin → sun-dried. | tunta phuti; soups | Lead-grey colour (darker than commercial tunta). Used primarily for household consumption. |
| Kaya — uma kaya | *uma kaya* | Oca (*Oxalis tuberosa*) — apilla ethnovariety group | Submerged in water for ~1 month → removed on evenings with anticipated frost → transferred to freezing site → frozen → sun-dried. | *uma kay phuti* (soaked, steam-cooked); also used in soups | Preferred variant among *kaya* preparations. Processing mirrors tunta method. |
| Kaya — juyphi kaya | *juyphi kaya* | Oca (*Oxalis tuberosa*) — apilla ethnovariety group | Frozen directly (no prior water immersion) → trampled underfoot to flatten → sun-dried. | *juyphi kay phuti* (soaked, pressed by hand, steam-cooked); also used in soups | Less preferred than *uma kaya.* Trampling flattens tubers before drying. |

### Supplementary Table. T. 2. Thermally Processed Grain and Legume

| Method | Aymara / local term | Base ingredient | Product name | Notes / use |
| --- | --- | --- | --- | --- |
| Toasting | *jamp'iña* | Maize | *tunqu jamp'i / tunqu aku* | Toasted whole *(jamp'i*) or ground into flour (*aku*); eaten immediately or used in soups, porridges, beverages |
| Toasting | *jamp'iña* | Barley | *siwar jamp'i / siwar aku* | Toasted whole or ground into flour; primary nourishment for elderly |
| Toasting | *jamp'iña* | Broad bean (Vicia faba) | *jawas jamp'i / jawas aku* | L:ikely heat reduces lectins and protease inhibitors; flour used in soups and other preparations |
| Toasting | *jamp'iña* | Peas (Lathyrus / Pisum) | *alwirj jamp'i / alwirj aku* | Heat reduces lectins and protease inhibitors |
| Toasting | *jamp'iña* | Quinoa (Chenopodium quinoa) | *aku* | Dry-roasted then ground; used in porridges (lawa), beverages (ullpu), soups (kaltu), breads (t'ant'a) |
| Toasting | *jamp'iña* | Cañihua (Chenopodium pallidicaule) | *aku* | Same processing pathway as quinoa |
| Toasting | *—* | Various (pit oven) | *kanka* | Roasting over embers; applied to meats (dried fish, beef, mutton); slow heat exposure |
| Boiling | *qhatiyaña* | Potato | *ch'uqi qhati* | Boiled whole with skin; eaten with soup or carried as packed meal (ququ) |
| Boiling | *qhatiyaña* | Oca | *apill qhati* | Boiled whole with skin |
| Boiling | *qhatiyaña* | Isaño | *isañ qhati* | Boiled whole with skin |
| Boiling | *qhatiyaña* | Potato (ritual) | *umar qhatita* | Small ritual potatoes boiled whole |
| Boiling | *mut'i* | Maize | *tunqu mut'i* | Soaked or unsoaked, boiled overnight; solid 'dry' accompaniment to soup |
| Boiling | *mut'i* | Broad bean | *jawas mut'i* | Soaked or unsoaked, boiled overnight |
| Boiling | *mut'i* | Pea | *alwirj mut'i* | Soaked or unsoaked, boiled overnight |
| Boiling | *allintat mut'i* | Maize | *allintat tunqu mut'i* | Pre-roasted before boiling to reduce cooking time |
| Boiling | *allintat mut'i* | Broad bean | *allintat jawas mut'i* | Pre-roasted before boiling to reduce cooking time |
| Boiling | *allintat mut'i* | Pea | *allintat alwirj mut'i* | Pre-roasted before boiling to reduce cooking time |
| Pit-roasting | *waja* | Potato | *ch'uqi waja* | Mashed or peeled after removal; served with clay sauce (phasa) or meat |
| Pit-roasting | *waja* | Oca | *apill waja* | Prepared in sealed earth oven on harvest days or post-ritual |
| Pit-roasting | *waja* | Isaño | *isañ waja* | Prepared in sealed earth oven |
| Pit-roasting | *waja* | Broad bean (pods) | *jawas waja* | Pods placed inside oven; sealed with soil |
| Pit-roasting | *waja* | Pea (pods) | *alwirj waja* | Pods placed inside oven; sealed with soil |
| Pit-roasting | *waja* | Meat / cheese | *kanka (meat)* | Placed in small clay pot inside oven; combined with tubers in communal/ceremonial context |
| Frying | *kaswiraña / thixiña* | Meat | *kaswira / thixi* | Oil or pork fat; includes dried fish, beef, mutton |
| Frying | *kaswiraña / thixiña* | Eggs | *kaswira / thixi* | Oil or pork fat |
| Frying | *kaswiraña / thixiña* | Qarasiña | *kaswira* | Oil or pork fat |
| Frying | *kaswiraña / thixiña* | Tortillas / fritters | *tortilla / fritter* | Oil or pork fat |
| Dehulling | *thijuta* | Barley | *siwar thiju* | Toasted then dehulled by rubbing in folded sheepskin; obsolete preparation made when no other food was available |
| Dehulling | *phata* | Barley (naked / q'ala siwara) | *phata (*pearled barley*)* | Soaked 30 min, hand-pounded with stone (piqaña), husk floated off (aytiraña), boiled; used in phata kaltu (soup) or as stew |

### Supplementary Table. T. 3. Diversity of Uses as food

| Dish / preparation | Aymara / local term | Records | Category | Base ingredient(s) | Description/use |
| --- | --- | --- | --- | --- | --- |
| ***Api*** | *api / apí* | 6 | Beverage | Purple maize flour; variants with yellow/white maize; sometimes added fruit | Hot thick warming beverage. Consumed as breakfast or snack. Low frequency; highly specific preparation. |
| ***Arroz*** | *arroz* | 15 | Introduced grain | Rice | Low-frequency Spanish-label category reflecting introduced staple. Signals gastronomic exchange. |
| ***Ch'uñu kaltu*** | *ch'uñu kaltu / ch'uñu allp kaltu* | 236 | Soup/broth | Ch'uñu (freeze-dried black potato), meat, onion, carrot, ají | Soup/stew with rehydrated ch'uñu as central ingredient; ch'uñu allp kaltu uses ch'uñu flour. Hearty, thickened broth. |
| ***Chuño / tunta* (dishes)** | *ch'uñu phuti / tunta phuti / chayru* | — | Freeze-dried tuber | Ch'uñu or tunta (rehydrated) | See freeze-drying section. Rehydrated products steam-cooked (phuti), incorporated into soups (chayru = chopped ch'uñu soup), or served as stew. |
| ***Cocido / caldo / sopa*** | *cocido, caldo, sopa* | 1,000+ | Soup/broth | Multiple taxa (potato, quinoa, maize, legumes, herbs) | Generic boiled soups and broths; the most frequent preparation mode overall. Primary vehicle for plant incorporation into daily meals. |
| ***Desayuno*** | *desayuno* | 41 | Meal context | Various | Breakfast as a categorisation label. Reflects a classification system that blends meal occasion with preparation type. |
| ***Ensalada*** | *ensalada* | 136 | Salad | Various (Spanish-language category) | Salad preparations. Spanish label reflects bilingual culinary naming and integration of mestizo dishes. |
| ***Frito*** | *frito* | 64 | Fried preparation | Various | Fried preparations as a category; blends technique with serving context. Includes meats, fritters, eggs. |
| ***Infusions*** | *mate / té* | 392 | Infusion | Various taxa (herbs, leaves) | Hot plant infusions consumed as beverages. Rival solid foods in frequency, reflecting the centrality of liquid nourishment in Aymara diet. |
| ***K'ispiña*** | *k'ispiña / mulu k'ispiña / q'api k'ispiña* | 121 | Quinoa preparation | Quinoa flour (aqallpu); limewater for saponin neutralisation | Limewater-kneaded dough steam-cooked in clay pot lined with ch'illiwa grass and salliwa leaves. Two forms: cylindrical cords (mulusu) and compressed palm-sized pieces (q'api). Cooled on inkuña cloth before eating. |
| ***Masticar (coca)*** | *masticar / acullicar* | 136 | Ritual / stimulant | Coca leaves (Erythroxylum spp.) | Coca leaf mastication classified as a form of food intake, not merely ritual or stimulant use. Mid-range frequency underscores cultural significance in Aymara dietary practice. |
| ***Munta / munta qhati*** | *munta / munta qhati* | 128 | Boiled tuber | Potato (Solanum spp.), ají amarillo / turmeric (pallallusa), onion, garlic | Peeled boiled potatoes in spiced yellow sauce. Highly valued in rural highlands; served at communal meals (quqawi/apthapi) with chuño, oca, or broad beans. Also packed for field meals. |
| ***Mut'i*** | *mut'i / tunqu mut'i / jawas mut'i / alwiri mut'i* | 112 | Boiled grain/legume | Maize (tunqu), broad bean (jawas), pea (alwiri) | Solid food boiled in water. Maize and broad bean often cooked together (energy + protein). Served alongside soup at home or carried as packed meal (ququ) during fieldwork. |
| ***P'isqhi*** | *p'isqhi* | 202 | Quinoa preparation | Quinoa (Chenopodium quinoa — desaponified) | Boiled unsalted quinoa seeds mixed with salt, consumed alone or with milk or cheese, or drowned in liquid. |
| ***Phiri*** | *aku phiri* | 159 | Flour preparation | Barley (aku/pitu); variant with cañihua (qañawa) | Boiling salted (or sugared) water cooled slightly then poured into aku; semi-dry consistency. Some recipes include oil. |
| ***Pitu / Aku*** | *pitu / aku / roasted flour* | 237 | Flour preparation | Barley, quinoa, cañihua, maize (toasted and milled) | Toasted grain milled into flour. Used to thicken soups, flavor dishes, or consumed standalone. Primary nourishment for elderly with limited dentition. |
| ***Qarasiña*** | *qarasiña* | 142 | Fried preparation | Quinoa flour; lime solution; oil or animal fat | Thin flat fritters. Dough prepared by skilled women using lime solution to neutralise saponins (assessed by colour and taste change). Circular forms require greater expertise than elongated ones. |
| ***Qhati*** | *qhati / chu'uqi qhati / apilla qhati / isañu qhati* | 373 | Soup/broth | Potato (Solanum spp.), oca (Oxalis tuberosa), isaño (Tropaeolum tuberosum) | Tubers cooked by boiling or steaming (~half each method), yielding named preparations per tuber species. Served at home or carried as packed meal (ququ). |
| ***Quisu umacha*** | *quisu umacha* | 50 | Dairy | Raw milk | Traditional fresh cheese made by naturally curdling raw milk. Soft, tangy, strong-smelling; consumed soon after preparation. Reflects mestizo / gastronomic exchange. |
| ***Raw consumption*** | *—* | 858 | Raw | Various taxa | Plants consumed with minimal or no processing; snacks, ritual foods, or accompaniments. Second most documented use overall. |
| ***Refresco*** | *refresco* | 45 | Beverage | Various | Cold beverage. Documented as a preparation category reflecting serving temperature/context rather than a single recipe. |
| ***Siwar aku*** | *siwar aku* | 7 | Flour preparation | Barley | Barley roasted flour; used like other aku types or to thicken soups. Low-frequency, specialized preparation. |
| ***Thayacha / jup'ucha*** | *thayacha / thächa / isañ thächa / apill thächa / ch'uqi qhat thächa / jup'ucha* | 190 | Freeze-dried tuber | Isaño (Tropaeolum tuberosum), oca, potato; also barley/cañihua aku for jup'ucha variant | Winter preparation: tubers or aku-dough discs boiled then left to freeze overnight on rooftop; consumed the following morning. Jup'ucha sweetened with sugar or isañu syrup. |
| ***Wallaqi (challwa kaltu)*** | *wallaqi / challwa kaltu / chawlla kaltu / challwa wallaqi* | 91 | Soup/broth | Fish (qarachi, Orestias agassizii and other Orestias spp.) from Lake Titicaca; herbs (Clinopodium bolivianum — muña/q'uwa) | Fish soup seasoned with strong aromatic herbs. Distinct from vegetable-based broths. |
| ***Wayk'a*** | *wayk'a / wayk'ani* | 237 | Condiment / stew | Chili (*ají)*; also onion, cumin, egg, or meat | *Wayk'a*: spicy sauce/condiment of chili and salt, served with solid dishes (ch'uñu phuti, qhati, mut'i). *Wayk'ani* (101 records): cooked stew incorporating *wayk'a* as a key spicy ingredient. |
